# Tumor-associated mesenchymal stromal cells modulate macrophage phagocytosis in stromal-rich colorectal cancer via PD-1 signaling

**DOI:** 10.1016/j.isci.2024.110701

**Published:** 2024-08-22

**Authors:** Niamh A. Leonard, Shania M. Corry, Eileen Reidy, Hannah Egan, Grace O’Malley, Kerry Thompson, Emma McDermott, Aoise O’Neill, Norashikin Zakaria, Laurence J. Egan, Thomas Ritter, Daniela Loessner, Keara Redmond, Margaret Sheehan, Aoife Canney, Aisling M. Hogan, Sean O. Hynes, Oliver Treacy, Philip D. Dunne, Aideen E. Ryan

**Affiliations:** 1 Discipline of Pharmacology and Therapeutics, School of Medicine, College of Medicine, Nursing and Health Sciences, University of Galway, Galway, Ireland; 2 Regenerative Medicine Institute (REMEDI), School of Medicine, College of Medicine Nursing and Health Sciences, University of Galway, Galway, Ireland; 3 Lambe Institute for Translational Research, School of Medicine, College of Medicine, Nursing and Health Sciences, University of Galway, Galway, Ireland; 4 Patrick G Johnston Centre for Cancer Research, Queen’s University Belfast, Belfast, Northern Ireland; 5 CÚRAM Centre for Research in Medical Devices, School of Medicine, College of Medicine, Nursing and Health Sciences, University of Galway, Galway, Ireland; 6 Centre for Microscopy and Imaging, Discipline of Anatomy, School of Medicine, College of Medicine, Nursing and Health Sciences, University of Galway, Galway, Ireland; 7 Barts Cancer Institute, Queen Mary University of London, London, UK; 8 Faculty of Engineering and Faculty of Medicine, Nursing and Health Sciences, Monash University, Melbourne, VIC, Australia; 9 Leibniz-Institut für Polymerforschung Dresden, Dresden, Germany; 10 Department of Colorectal Surgery, Galway University Hospital, Galway, Ireland; 11 Division of Anatomical Pathology, Galway University Hospital, Galway, Ireland; 12 Discipline of Pathology, School of Medicine, College of Medicine, Nursing and Health Sciences, University of Galway, Galway, Ireland; 13 Cancer Research UK Beatson Institute, Glasgow, UK

**Keywords:** Immunology, Cancer, Transcriptomics

## Abstract

CMS4 colorectal cancer (CRC), based on the consensus molecular subtype (CMS), stratifies patients with the poorest disease-free survival rates. It is characterized by a strong mesenchymal stromal cell (MSC) signature, wound healing-like inflammation and therapy resistance. We utilized 2D and 3D *in vitro, in vivo*, and *ex vivo* models to assess the impact of inflammation and stromal cells on immunosuppression in CMS4 CRC. RNA sequencing data from untreated stage II/III CRC patients showed enriched TNF-α signatures in CMS1 and CMS4 tumors. Secretome from TNF-α treated cancer cells induced an immunomodulatory and chemotactic phenotype in MSC and cancer-associated fibroblasts (CAFs). Macrophages in CRC tumours migrate and preferentially localise in stromal compartment. Inflammatory CRC secretome enhances expression of PD-L1 and CD47 on both human and murine stromal cells. We demonstrate that TNF-α-induced inflammation in CRC suppresses macrophage phagocytosis via stromal cells. We show that stromal cell-mediated suppression of macrophage phagocytosis is mediated in part through PD-1 signaling. These data suggest that re-stratification of CRC by CMS may reveal patient subsets with microsatellite stable tumors, particularly CMS4-like tumors, that may respond to immunotherapies.

## Highlights

• CMS-4 associated TNF-α induced inflammation increases macrophage recruitment
• Inflammatory tumor secretome induces stromal cell expression of PD-L1, PD-L2 and CD47
• Inflammation induces stromal-mediated suppression of macrophage phagocytosis
• Targeting PD-L1/PD-1 in stromal-macrophage cultures enhances macrophage phagocytosis

## Graphical abstract

**Figure:**
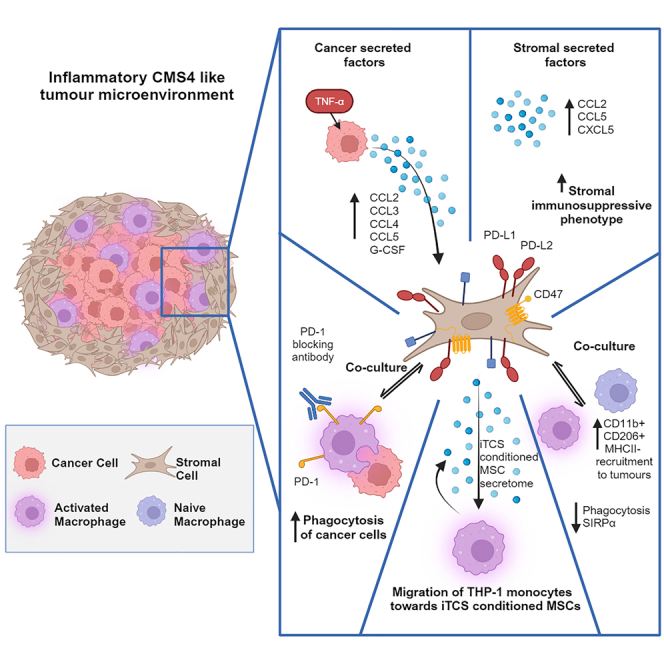

## Introduction

Colorectal cancer (CRC) is the third most common cancer worldwide and despite improvements in screening and treatment options, it remains the second leading cause of cancer-related deaths.^1^^,^^2^ The high mortality rate is partly attributed to increasing incidence, late diagnosis, and a lack of effective treatments for advanced disease.^3^^,^^4^ It is now widely accepted that in addition to epithelial cancer cells, the tumor microenvironment (TME), including mesenchymal stromal (MSCs) and immune cells, wields a profound influence on patient responses to treatments and contributes to treatment resistance.^5^^,^^6^^,^^7^

The consensus molecular subtypes (CMS) classification system emerged as a valuable tool and divides CRC based on transcriptional signature into four distinct subtypes, CMS1-4, with prognostic significance for survival rates and treatment response.^8^^,^^9^ Patients with CMS4 tumors comprise 23% of all cases and are characterized by a high number of MSCs including cancer-associated fibroblasts (CAFs), and a wound healing-like inflammatory signature that is associated with poor prognosis and high relapse rates.^8^^,^^10^ Histology-based stromal classifiers strongly correlate with established transcriptional signatures in CMS4 tumors, enabling classification from tumor sections.^11^ CMS4 patients have limited benefits from surgery and/or chemotherapy, therefore alternative treatment options are urgently needed.^12^ Understanding the role of inflammation and stromal cell crosstalk in the CRC TME is critical to inform the design of more targeted and effective treatments for stromal-rich CMS4 tumors.

Immunotherapies, particularly immune checkpoint inhibitors (ICIs), have revolutionized cancer treatment and in some cases have substantially improved survival rates across many cancer types. However, the use of ICI in CRC is currently limited to patients with microsatellite instability (MSI)-high tumors, which mainly fall within the CMS1 subtype due to their high mutational burden and infiltration of cytotoxic T cells.^13^^,^^14^ While CMS2 and CMS3 tumors do not have a large immune infiltrate, they have the most beneficial response to adjuvant chemotherapy^12^ in contrast to CMS4 tumors, which respond poorly. The TME in CMS4 tumors is rich in myeloid cells,^13^ with macrophages reported to comprise 50% of immune cell population in some CRC TMEs,^15^^,^^16^ suggesting a potential therapeutic benefit of innate immune cell-focused immunotherapies. Recent data suggests that SIRP-α and PD-1 receptors modulate macrophage anti-tumor function through engagement with their respective ligands, CD47 and PD-L1/PD-L2.^17^^,^^18^ We and others have also shown that stromal cells and CAFs express immunomodulatory ligands that orchestrate an immunosuppressive TME.^19^^,^^20^^,^^21^^,^^22^

Tumor-associated macrophages (TAMs) predominantly originate in the bone marrow as monocytes, and tumor-derived inflammation recruits TAMs to the tumor site.^23^^,^^24^^,^^25^ CAFs in CRC attract monocytes to the TME and enrich intra-tumoral TAM phenotypes.^26^ TAMs are often identified by CD11b and CD68, with markers such as IFN-γ and HLA-DR associated with an anti-tumor phenotype, while CD163, CD206, PD-L1 and ARG1 are associated with a tumor-promoting phenotype.^15^^,^^27^^,^^28^^,^^29^ Targeting TAMs may inhibit their pro-tumorigenic functions, such as promoting cancer growth, neo-vascularization, invasion, tissue remodeling and immunosuppression.^30^ Additionally, TAMs recruit and respond to other immune cells in the TME,^31^ recognize damage-associated molecular patterns (DAMPs), phagocytose cancer cells and present tumor-associated antigens, orchestrating an anti-tumor immune response.^30^ However, the role of inflammation in the stromal cell – macrophage crosstalk is not fully understood.

In this study, we investigate the mechanisms of inflammation induced immunomodulation of stromal cells in the TME of CRC. Using *in vitro*, *in vivo* and *ex vivo* functional assays, we show that TNF-α-mediated inflammation, a feature of CMS4 tumors, enhances the immunosuppressive stromal cell-macrophage crosstalk and induces macrophage recruitment. We found that inflammatory tumor cell secretome (iTCS) induces PD-L1, CD47 and PD-L2 expression on MSCs and CAFs. TCS and iTCS-conditioned MSCs suppress macrophage-mediated phagocytosis, a key anti-tumor function. Utilizing a stage II/III untreated cohort, we demonstrated a correlation of expression of macrophage-related markers (CCL-2 and CD11b) and immunosuppressive markers (PD-1) with a poor prognosis across all samples, as well as specifically within CMS4 samples. We establish that targeting PD-L1/PD-1 signaling in stromal cell-macrophage co-cultures reverses the suppression of phagocytosis and enhances macrophage anti-tumor effector functions. These data suggest the potential of targeting these pathways to reverse the suppression of macrophage anti-tumor functions using immunotherapies for stromal-rich CMS4 tumors.

## Results

### TNF-α inflammatory signaling is enhanced in CMS4 tumors

**Figure 1:**
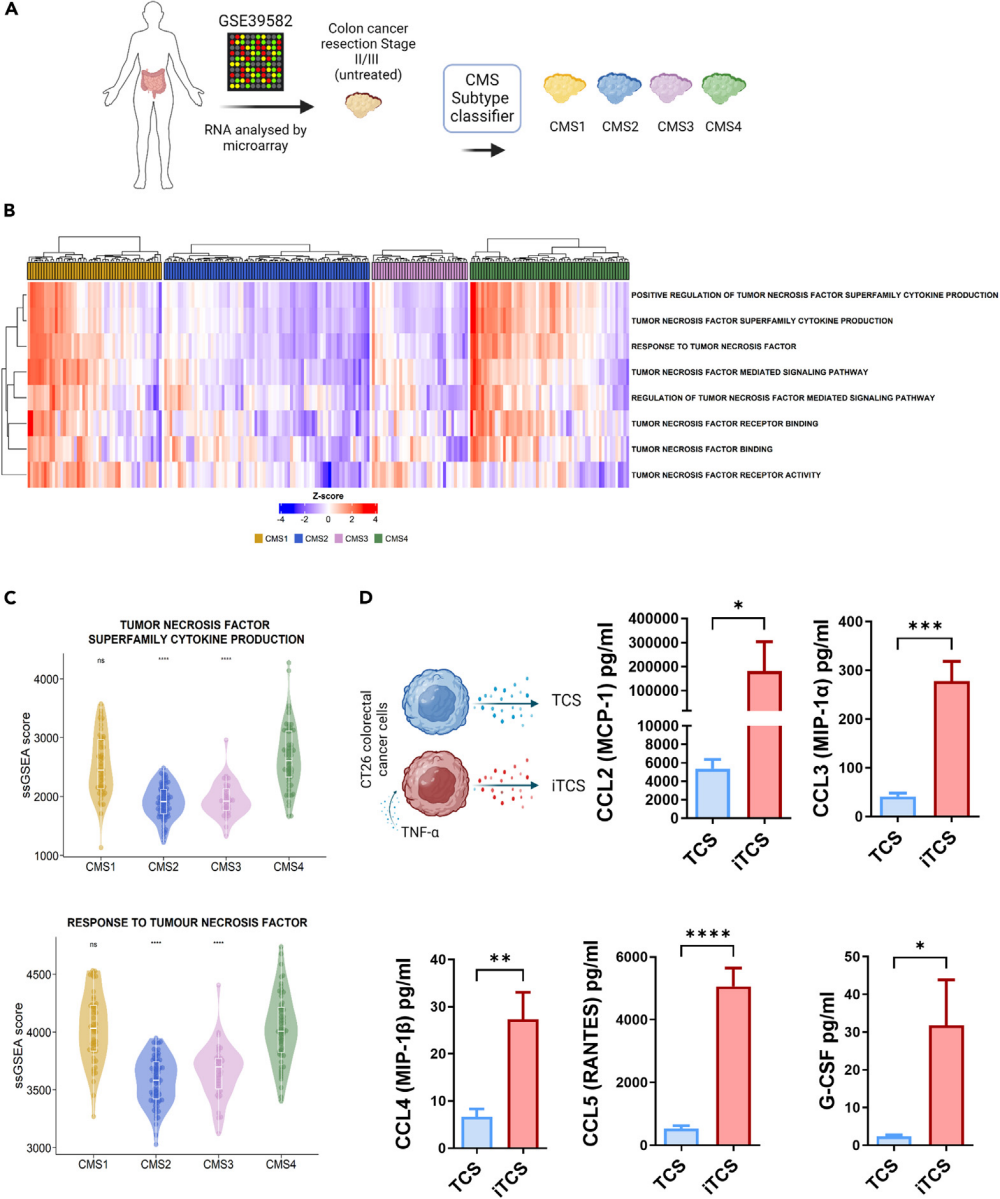
TNF-α signaling in CMS4 tumors alters cancer cell secretory phenotype (A) Transcriptional profiles of stage II/III untreated colon cancer samples (GSE39582) were retrieved and CMS classified (*n* = 258; CMS1 = 49, CMS2 = 75, CMS3 = 35, CMS4 = 58, unknown = 41). (B) Heatmap of single sample gene set enrichment analysis (ssGSEA) scores of pathways related to TNF-α production and signaling. (C) ssGSEA analysis of pathways related to TNF-α production (top) and signaling (bottom) (Wilcoxon rank-sum test, CMS4 as reference group). (D) Experimental outline for generating tumor cell secretome (TCS) and inflammatory tumor cell secretome (iTCS). CT26 cells, a Balb/c colon cancer cell line, was cultured for 72 h in the presence (iTCS) or absence (TCS) of TNF-α. TNF-α was added 24 h prior to the collection of media to generate iTCS and TCS, respectively (top left). Bioplex analysis of secreted factors CCL2, CCL3, CCL4, CCL5 and G-CSF in the TCS or iTCS from CT26 cells. Error bars, mean ± SEM; ∗, *p* < 0.05; ∗∗, *p* < 0.01; ∗∗∗, *p* < 0.001 ∗∗∗∗*p* < 0.0001 by Wilcoxon rank-sum test, CMS4 as reference group (C) or unpaired t-test (D). n = 2–6.

Patients with CMS4 tumors have the lowest disease-free survival rates and are characterized by a high density of MSCs and an inflammatory signature.^8^^,^^10^ To understand how inflammation and stromal cells contribute to immunomodulation in the TME, we sought to develop a more representative cell culture system to model the CMS4-like TME. TNF-α is reported to be increased in over 80% of all CRC patients, positively correlates with increased disease stage,^32^^,^^33^ and forms a mechanistic link between inflammation and cancer. To analyze the enrichment of TNF-α signaling pathways across different CRC molecular subtypes, we utilized transcriptional profiles from an expression dataset (GSE39582) comprising untreated stage II/III colon cancer tumors (*n* = 258)^34^ as outlined in Figure 1A. Individual samples were assigned a CMS classification and single sample gene set enrichment scores (ssGSEA) for pathways involved in TNF-α production, receptor binding, response and signaling; all of which were observed to be enriched in CMS1 and CMS4 tumors compared to CMS2 or CMS3 tumors (Figure 1B). ‘Tumor necrosis factor superfamily cytokine production’ and ‘Response to tumor necrosis factor’ enrichment scores were significantly higher in CMS4 compared to CMS2 or CMS3 and comparable to CMS1 (Figure 1C). To analyze the biological effects of TNF-α in CRC, we generated tumor cell secretome (TCS) and inflammatory TCS (iTCS) by treating colorectal cancer cells with TNF-α (Figure 1D). TNF-α induced a significant increase in the secretion of chemokines and cytokines from CT26 cancer cells including CCL2, CCL3, CCL4, CCL5 and G-CSF (Figure 1D). These factors have been implicated in the support of colon cancer growth, chemotaxis of stromal cells, metastasis, and regulation of immune cell function.^35^^,^^36^^,^^37^ This data demonstrates that TNF-α, which is associated with the CMS4 inflammatory signature, induced secretion of pro-tumor molecules and stromal cell chemo attractants from tumor cells.

### TNF-α inflammation induces an immunomodulatory stromal cell phenotype

**Figure 2:**
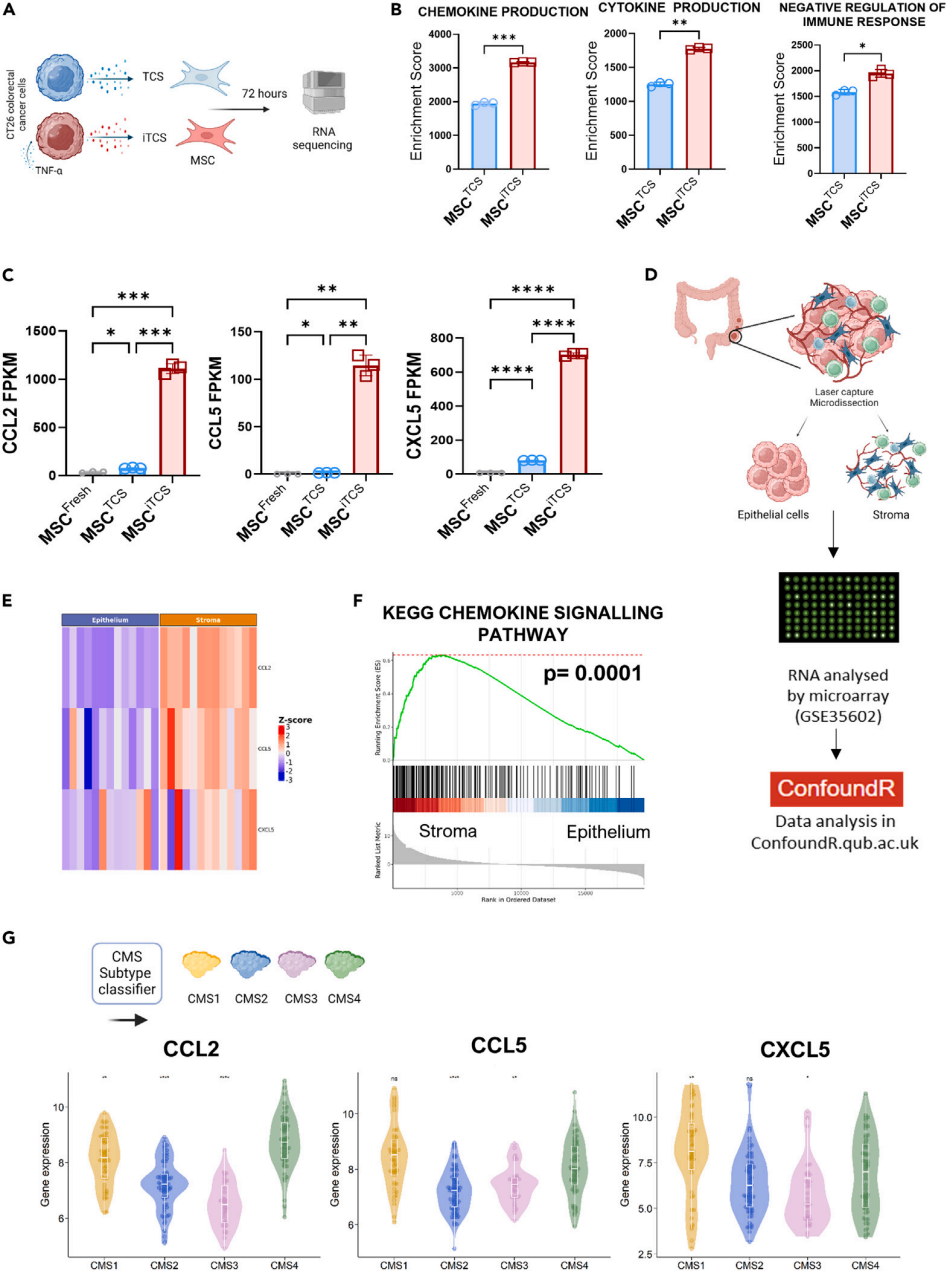
Inflammatory tumor secretome induces MSC immunomodulatory and chemotactic phenotypes (A) Experimental outline of MSC conditioning with TCS and iTCS. (B) Gene set enrichment analysis (GSEA) conducted on RNA-seq data of CT26 TCS and iTCS-treated mMSCs (*n* = 3). (C) Fragments per kilobase of exon per million mapped fragments (FPKM) values for *CCL2*, *CCL5* and *CXCL5* in control and tumor-conditioned MSC groups (*n* = 3). (D) Transcriptional profiles of laser capture microdissected CRC samples (GSE35602), were analyzed using ConfoundR application (https://confoundr.qub.ac.uk/). (E) Expression heatmap of chemoattractant gene transcripts (*CCL2, CCL5 and CXCL5*) in the stromal and epithelial compartments of CRC tumors (*n* = 13) (GSEA35602). (F) KEGG pathway analysis of chemokine signaling pathway. (G) Gene expression levels of *CCL2, CCL5,* and *CXCL5* across CMS subtype (GSE39582) (Wilcoxon rank-sum test, CMS4 as reference group). Error bars, mean ± SD; ∗, *p* < 0.05; ∗∗, *p* < 0.01; ∗∗∗, *p* < 0.001; ∗∗∗∗, *p* < 0.0001 by paired t-test (B) or one-way ANOVA and Tukey post hoc test (C) or Wilcoxon rank-sum test, CMS4 as reference group (G).

Next, we investigated the ability of TCS and iTCS to influence tumor-associated stromal cell immunomodulatory phenotype (Figure 2A). Murine bone marrow-derived mesenchymal stromal cells (mMSCs) were conditioned with TCS or iTCS for 72 h, cells were harvested and RNA-Seq analysis was performed (Figure 2A). Gene set enrichment analysis (GSEA) revealed that stromal cells treated with TCS had significantly higher enrichment scores for genes related to cytokine and chemokine production (Figure 2B). The inflammatory tumor-conditioned mMSCs also had a significantly higher enrichment score for the negative regulation of immune response pathway compared to TCS-treated mMSCs (Figure 2B). Next, we investigated the expression of individual genes involved in innate and adaptive immune cell recruitment (*CCL2*, *CCL5* and *CXCL5*; Figure 2C). We observed that these genes were expressed at significantly higher levels in the iTCS-treated mMSCs compared to TCS-treated mMSCs or baseline expression levels (Figure 2C).

To validate the transcriptional signatures in human tumor-associated stromal cells, we next assessed the expression of chemokines and cytokines using ConfoundR.^38^^,^^39^^,^^40^ ConfoundR (https://confoundr.qub.ac.uk/) enables the visualization of the selected signaling pathways in the epithelium and stromal compartments across CRC samples (GSE35602) (Figure 2D).^41^ We found that expression of *CCL2* and *CCL5* genes were higher in the stromal compartment than in the epithelium in most patients (Figures 2E and S1A). GSEA (using ConfoundR) between the two compartments using Kyoto Encyclopedia of Genes and Genomes (KEGG) and Hallmark gene sets, highlighted that the chemokine signaling pathway was significantly enriched in the stromal versus epithelial compartment, as well as cytokine receptor interactions, inflammatory response and TNF-α signaling pathways (Figures 2F and S1B).

As this dataset was not CMS profiled, we next investigated the differences in gene expression across the CMS subtypes in the GSE39582 dataset (Figures 1 and 2G). *CCL2* mRNA expression was significantly higher in CMS4 tumors compared to other subtypes, while *CCL5* levels were comparable in CMS4 and CMS1 tumors but significantly higher than CMS2 or CMS3 tumors (Figure 2G). In contrast, *CXCL5* expression was higher in CMS1 compared to CMS4 and lower in CMS3 compared to CMS4 (Figure 2G). Together this data demonstrates that the inflammatory tumor microenvironment in CMS4 tumors may impact stromal cell chemokine and immunomodulatory transcriptional profiles. Enhanced secretion of chemotactic factors may suggest the potential for enhanced recruitment and immunomodulation in the CRC TME.

### *In vitro* 3D model of colorectal cancer and MSCs recapitulate CMS4 characteristics

**Figure 3:**
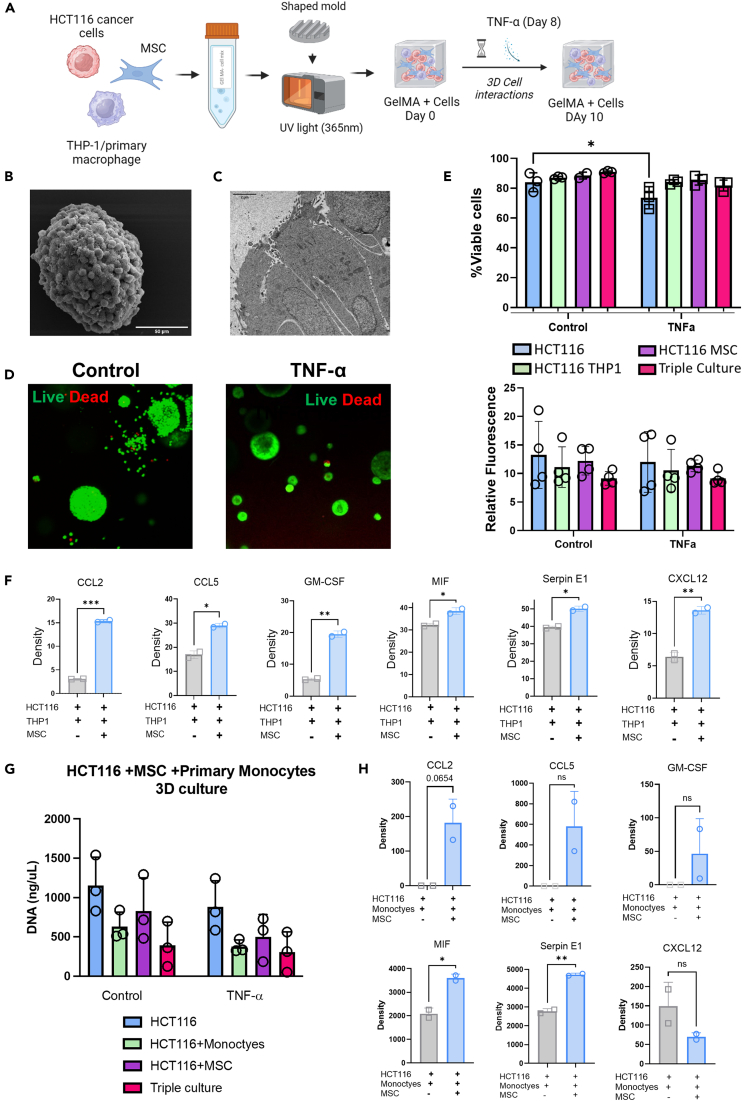
MSCs alter the secretory phenotype toward macrophage modulation and tumor promotion in a 3D CMS4-like culture system (A) Experimental outline depicting how the human 3D model of CMS4 CRC was generated by combining CRC cells, human bone marrow-derived MSCs and THP1 monocytes in a GelMA hydrogel for 10 days, with TNF-α addition at day 8 once complex spheroids had formed. (B) Representative scanning electron microscope image of a spheroid isolated from a triple cells 3D culture, scale bar = 50 μm. (C) Representative transmission electron microscope image of cells as part of the multicellular spheroid, scale bar = 2 μm. (D) Confocal microscopy images of Calcein AM (live) and Propidium Iodide (dead) stained gels, 10× magnification, of control and TNF-α treated samples. (E) Percentage cell viability of dissociated spheroids stained with Sytox blue and analyzed by flow cytometry (*n* = 3) (top). Metabolic activity of the culture systems measured by Alamar blue and displayed as relative fluorescence at day 10 relative to day 1 (*n* = 4) (bottom). (F) The secretome from the TNF-α treated 3D CRC culture system with or without MSCs was analyzed using a Proteome Profiler Human Cytokine Array Kit. Data from Human Cytokine arrays are expressed as pixel density of spots on the cytokine array membrane (*n* = 2). (G) Proliferation of HCT116, hMSCs and primary monocytes in GelMA hydrogel +/− TNF-α for 10 days analyzed using CyQuant analysis kit. (H) The secretome from the TNF-α treated 3D CRC culture system with primary monocytes with or without MSCs was analyzed using a Proteome Profiler Human Cytokine Array Kit. Data from Human Cytokine arrays are expressed as pixel density of spots on the cytokine array membrane (*n* = 2). Error bars, mean ± SD; ∗, *p* < 0.05, ∗∗, *p* < 0.01; ∗∗∗, *p* < 0.001 by two-way ANOVA and Tukey post hoc test (E) or paired t-test (F and H).

To determine the immunomodulatory roles of stromal cells in a three-dimensional (3D) tumor microenvironment, we developed an *in vitro* 3D multicellular human CMS4-like model (Figure 3A). HCT116 CRC cells, hMSCs and THP-1 monocytes or primary monocytes were cultured in GelMA^42^ as illustrated in Figure 3A. Multicellular spheroids were formed when the cells were cultured for over 10 days (Figure 3B). Transmission electron microscopy revealed that cells in the spheroids acquired a polarized phenotype similar to that seen in epithelial cells in the colon (Figure 3C). In addition to the incorporation of three different cell types, TNF-α was added to mimic the CMS4-like inflammatory signature. Cell viability in the 3D culture system was not affected by the addition of TNF-α, as shown by fluorescent microscopy (Figure 3D) and flow cytometry (Figure 3E (top)). Cell metabolic activity was maintained in the TNF-α-stimulated 3D cell cultures (Figure 3E (bottom)). Similar observations were seen with primary monocytes in 3D culture with HCT116 and MSC. CyQUANT analysis revealed that TNF-α did not significantly alter proliferation of cells in the GelMA (Figure 3G). Together these findings demonstrate that the GelMA hydrogel supports the viability of multicellular CMS4-like 3D culture systems.

Next, we profiled the secretome of the 3D cell cultures with or without the inclusion of stromal cells and TNF-α. The addition of hMSCs to the 3D culture with THP-1 cells and HCT116 cancer cells increased the release of chemokines that recruit both innate and adaptive immune cells (e.g., CCL2 and CCL5), factors that alter cell differentiation and function in myeloid cells (e.g., GM-CSF, MIF) and proteins that have direct and indirect tumor-promoting effects (e.g., SerpinE1 and CXCL12) (Figure 3F). Similar trends were observed in the 3D co-cultures with primary monocyte cells, hMSC and HCT116 cancer cell lines where there was a trend increase in CCL2, CCL5 and GM-CSF in cultures with the hMSCs. A significant increase was observed in secretion of MIF and SerpinE1 (Figure 3H). These data demonstrate that stromal cells alter the 3D tumor-secretory phenotype and contribute to the immunomodulatory landscape of CMS4 tumors in physiologically relevant multicellular 3D environments.

### Macrophages are enriched in stromal-rich CRC and localize in the stromal compartment

Macrophage infiltration in CRC tumors has been reported to have conflicting prognostic value. Unlike many other tumors where a high macrophage infiltrate is associated with worse prognosis and treatment response, in CRC, there have been reports of macrophages both promoting^43^^,^^44^ and inhibiting tumor progression and therapeutic response.^45^^,^^46^ This highlights that understanding their localization, phenotype and function in the TME could hold prognostic value in CRC.

**Figure 4:**
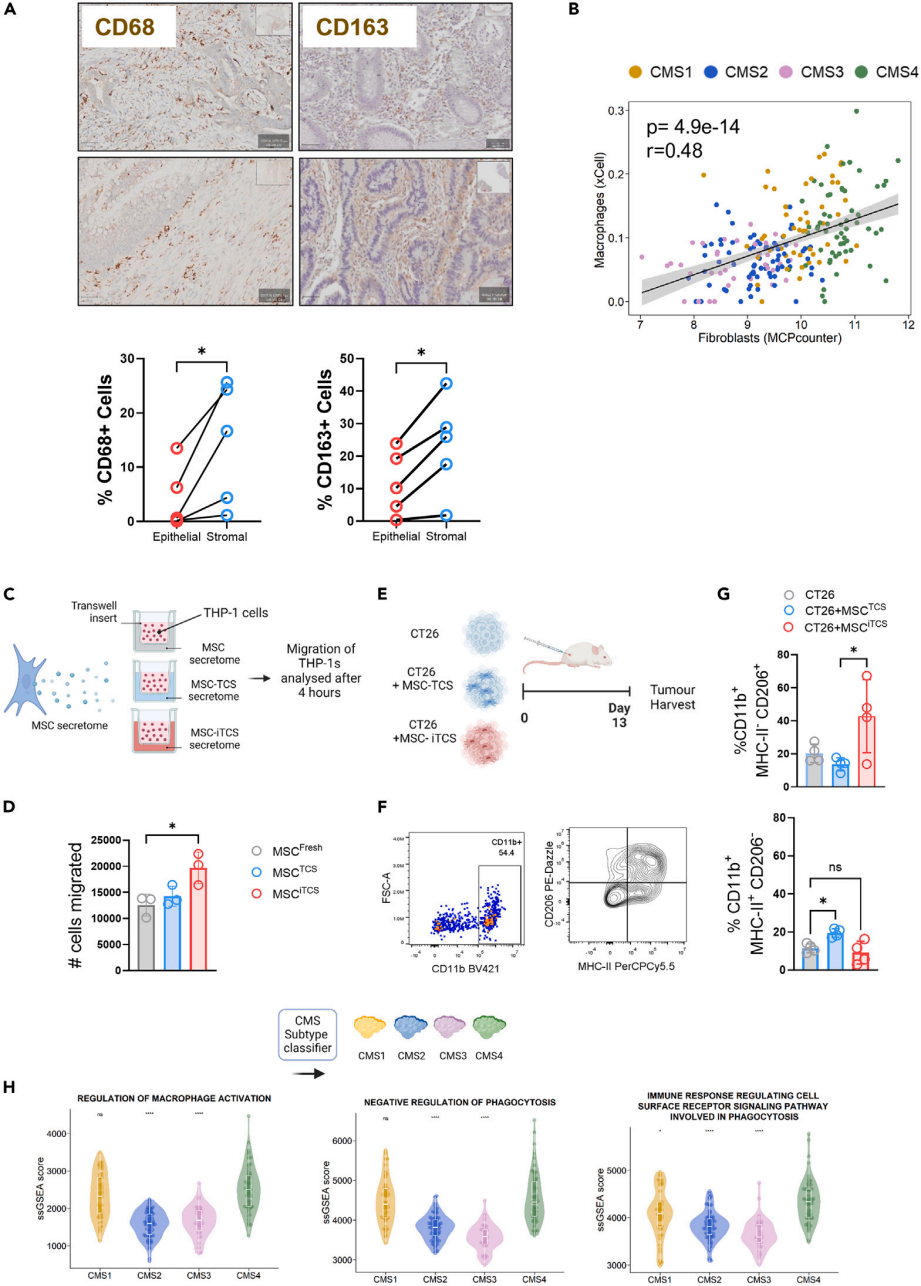
Macrophages in CRC tumors migrate and preferentially localise in the stromal compartment and positively correlate with fibroblast score (A) CD68 and CD163 staining of human CRC tissue, scale bar = 50 μm. Quantification of the percentage CD68^+^ and CD163+ cells in the epithelial or stromal compartment of the tumor (*n* = 5 patient samples) (below). (B) Pearson’s correlation between cell population estimates for fibroblasts and macrophages provided by MCPcounter and xCell, respectively (GSE39582) (colored by CMS). (C) Experimental outline. Transwell migration assay assessing THP-1 migration toward conditioned media from treated human MSCs. (D) Number of THP-1 cells migrated through the Transwell insert. (E) Experimental outline of murine tumor model. Balb/c mice were injected subcutaneously in the right flank with either CT26 cells alone, CT26 + TCS treated mMSCs or iTCS mMSCs. Tumors were harvested 13 days post-injection. (F) Flow cytometry gating strategy used to analyze single, live, CD45^+^, CD11b^+^, MHC-II^+/−^ CD206^+/−^ cells. (G) Percentage of CD11b+MHC-II^-^CD206^+^ (top) or CD11b+MHC-II^+^CD206^-^ (bottom) cells in murine tumors. (H) ssGSEA of pathways related to macrophage activation (left), phagocytosis (middle) and cell surface receptor signaling pathways involved in phagocytosis (right) (GSE39582). Error bars, mean ± SEM; ∗, *p* < 0.05; ∗∗∗, *p* < 0.001; ∗∗∗∗, *p* < 0.0001 by paired t-test (A), one-way ANOVA and Tukey post hoc test (D and G) or Wilcoxon rank-sum test, CMS4 as reference group (H).

To assess the spatial distribution of macrophages within the CRC TME, FFPE slides from CRC patient surgical resections were assessed for CD68 and CD163 expression. Stromal and epithelial compartments were distinguished as shown in Figure S2A. We observed significantly more CD68^+^ macrophages (Figure 4A, left) and CD163+ macrophages (Figure 4A, right) in the stromal compartment than the epithelial regions (Figures 4A, S2A, and S2B) and the frequency of macrophage infiltration varied between patients. This observation was supported by the significant positive correlation between cell population transcriptional estimates of macrophages and fibroblasts (Figure 4B). Together this data demonstrates that CD68^+^ and CD163+ macrophages localize in the stromal compartment and may represent an immunotherapeutic target in stromal-rich CMS4-like tumors.

To validate the chemotactic effects of tumor-conditioned stromal cells, we used a transwell migration assay to measure monocyte chemotaxis (Figure 4C). THP-1 monocytes show significantly enhanced migration toward condition media from iTCS-treated MSCs compared to conditioned media from unconditioned MSCs (Figure 4D). To further validate this finding, we assessed macrophage infiltration in subcutaneous tumors in immunocompetent mice using CT26 cancer cells with and without co-injection of conditioned-MSCs as illustrated in Figure 4E to model CMS4-like tumors. Tumors were harvested on Day 13 and analyzed by flow cytometry for CD11b+, MHC-II^+/−^/CD206^+/−^ macrophages (Figure 4F). iTCS MSC co-injected tumors had a significantly higher frequency of macrophages, specifically TAMs (CD11b^+^MHC-II^-^ CD206^+^) compared to TCS-treated MSCs and control CT26 tumors (Figure 4G (top)). TCS MSC, CT26 co-injected tumors had a higher frequency of CD11b^+^MHC-II^+^ CD206^-^ macrophages (Figure 4G (bottom)). These data provides evidence for enhanced recruitment of macrophages of both pro-and anti-inflammatory phenotypes to the CRC TME of stromal-rich tumors. The data also suggests that specific macrophage phenotypes may be influenced by inflammatory stromal cell signaling in the TME.

To assess transcriptional signatures relating to macrophages in CRC, we assessed gene set enrichment scores for a variety of signatures involved in the activation or regulation of macrophage functions across CMS subtypes (Figure S3A). CMS1 and CMS4 tumors had higher enrichment scores for macrophage-related pathways than CMS2 or CMS3 tumors (Figure S3A). CMS4 tumors had significantly higher enrichment scores for signatures related to the regulation of macrophage activation and negative regulation of phagocytosis when compared to CMS2 or CMS3 tumors, but comparable levels to CMS1 tumors (Figure 4H). A transcriptional signature of immune response regulating cell surface receptor signaling pathways involved in phagocytosis was significantly increased in CMS4 tumors compared to the other three CMS subtypes (Figure 4H). The data suggests that CMS4 tumors alter macrophage activation and anti-tumor functions, such as phagocytosis. This suggests that it may be functionally important to assess and to target receptor/ligand interactions involved in the regulation of phagocytosis between stromal cells and macrophages.

### Inflammatory tumor secretome induces stromal cell expression of PD-L1 and CD47

**Figure 5:**
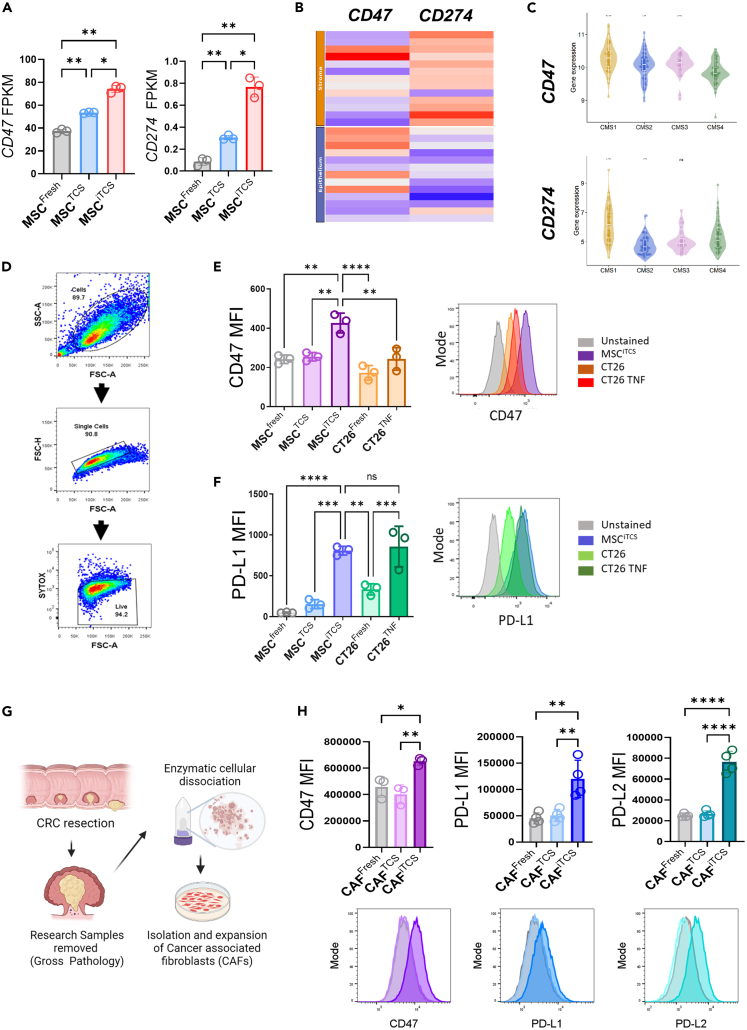
Inflammatory CRC secretome induces phagocytosis inhibitory molecules PD-L1, PD-L2 and CD47 on stromal cells (A) FPKM values for *CD47* and *CD274* in control and tumour-conditioned MSC groups (*n* = 3). (B) Expression heatmap of *CD47* and *CD274* (PD-L1) in the stromal and epithelial compartments of human CRC samples (*n* = 13) created by ConfoundR (GSE35602). (C) Gene expression of *CD47* and *CD274* (PD-L1) (GSE39582), grouped according to CMS molecular subtypes. (D) Flow cytometry gating strategy used to analyze single, live stromal cells. (E) CD47 expression analyzed by flow cytometry on Balb/c MSCs and CT26 CRC cells. Data displayed as median fluorescent intensity (MFI) with representative histograms (right) (*N* = 3). (F) PD-L1 expression analyzed by flow cytometry on Balb/c MSCs and CT26 CRC cells displayed as MFI with representative histograms (right) (*n* = 3). (G) Experimental outline showing isolation of cancer associated fibroblasts (CAFs) from primary CRC samples. (H) CD47, PD-L1 and PD-L2 expression on CAFs isolated from human CRC tumors, treated with HCT116 TCS or iTCS, analyzed by flow cytometry and displayed as MFI (*n* = 4 patient samples). Error bars, mean ± SD; ∗, *p* < 0.05; ∗∗, *p* < 0.01; ∗∗∗, *p* < 0.001; ∗∗∗∗, *p* < 0.0001 by one-way ANOVA and Tukey post hoc test (A, E, F, and H) or Wilcoxon rank-sum test, CMS4 as reference group (C).

Following the identification of signatures associated with the regulation of phagocytosis enriched in CMS4 tumors, we sought to investigate the expression of CD47 and CD274 (PD-L1), two immunomodulatory ligands that regulate macrophage phagocytosis.^17^^,^^18^ RNA-seq analysis of conditioned-mMSC revealed that MSCs treated with iTCS significantly increased their expression of CD47 and CD274 (Figure 5A). Using ConfoundR, we assessed the transcriptional expression of CD47 and CD274 in the stromal vs. epithelial areas of CRC (GSE35602) (Figures 5B and S4A). CD47 expression varied between samples and tumor compartment (Figures 5B and S4A). In comparison, CD274 expression was higher in the stromal compartment compared to the epithelial compartment in CRC (Figures 5B and S4A (right)). In relation to CMS4, it was observed that mRNA expression of *CD47* was low in CMS4 tumors (Figure 5C, upper), whereas *CD274* expression was highest in CMS1 tumors, and CMS4 tumors had significantly more *CD274* than CMS2 tumors (Figure 5C, lower).

We next sought to assess the expression of these immunomodulatory molecules on tumor-conditioned stromal cells by flow cytometry (Figure 5D). Expression of CD47 was induced at significantly higher levels on the iTCS-treated mMSCs compared to the TCS-treated or control mMSCs (Figure 5E). Conditioning of the MSCs with heat-inactivated iTCS reduced CD47 expression, suggesting that the effect was mediated by factors secreted by tumor cells and not due to nutrient deprivation (Figure S5A). In addition, treatment of mMSC with TNF-α at the levels found in the iTCS at the point of collection demonstrates that the induction was not caused by residual TNF-α and must be attributed to an active molecule produced by the inflammatory tumor cells (Figure S5A). CD47 was not significantly induced in CRC cells treated with TNF-α (Figure 5E), suggesting the effect is specific to stromal cells. iTCS-induced expression of PD-L1 in the stromal cells (Figure 5F) was mediated by factors actively secreted by cancer cells (Figure S5B). In contrast to CD47, TNF-α induced an increase in PD-L1 expression on tumor cells, however, the level was comparable to that seen on the iTCS-treated MSCs (Figure 5F). The induction of CD47 and PD-L1 by iTCS were validated in a human model system. TCS and iTCS was generated from two human CRC cell lines, HT29 and HCT116, representing both MSS and MSI features, respectively. iTCS from both cell lines induced significantly higher expression of cell surface CD47 and PD-L1 on MSCs than TCS conditioning (Figure S6A).

To validate these findings in primary human CRC stromal cells, we isolated CAFs from CRC patient samples following surgical resection and expanded them *ex vivo* (Figure 5G). The isolated CAFs were characterized by flow cytometry for CD45^−^ EpCAM^−^ and CD90^+^, FAP+ and Podoplanin+ expression (Figure S6B). CAFs expressed each of the positive CAF markers at high levels following isolation except for PDGFR-α which showed low baseline expression (Figure S6B). However, following the conditioning of CAFs with HCT116 iTCS, we observed the induction of PDGFR-α and increased expression of Podoplanin. (data not shown). This data demonstrates that inflammatory tumor secretome restored CAF phenotype after extended *ex vivo* culture.^47^^,^^48^ iTCS-treated CAFs showed increased expression of CD47 and PD-L1 similar to observations in the mouse model system. There was also an increased expression of PD-L2, which also binds to the PD-1 receptor, on CAFs in response to the inflammatory tumor secretome (Figure 5H (right)). We found no secreted PD-L1 in the conditioned media of the TCS or iTCS-treated CAFs or MSCs and control groups (data not shown). These experiments show that the induction of a stromal cell immunomodulatory phenotype is reproducible across species and MSC/CAF sources following tumor conditioning. Our findings demonstrate that inflammation alters the CRC secretome and potentiates an immunomodulatory stromal cell phenotype characterised by the expression of CD47, PD-L1 and PD-L2.

**Figure 6:**
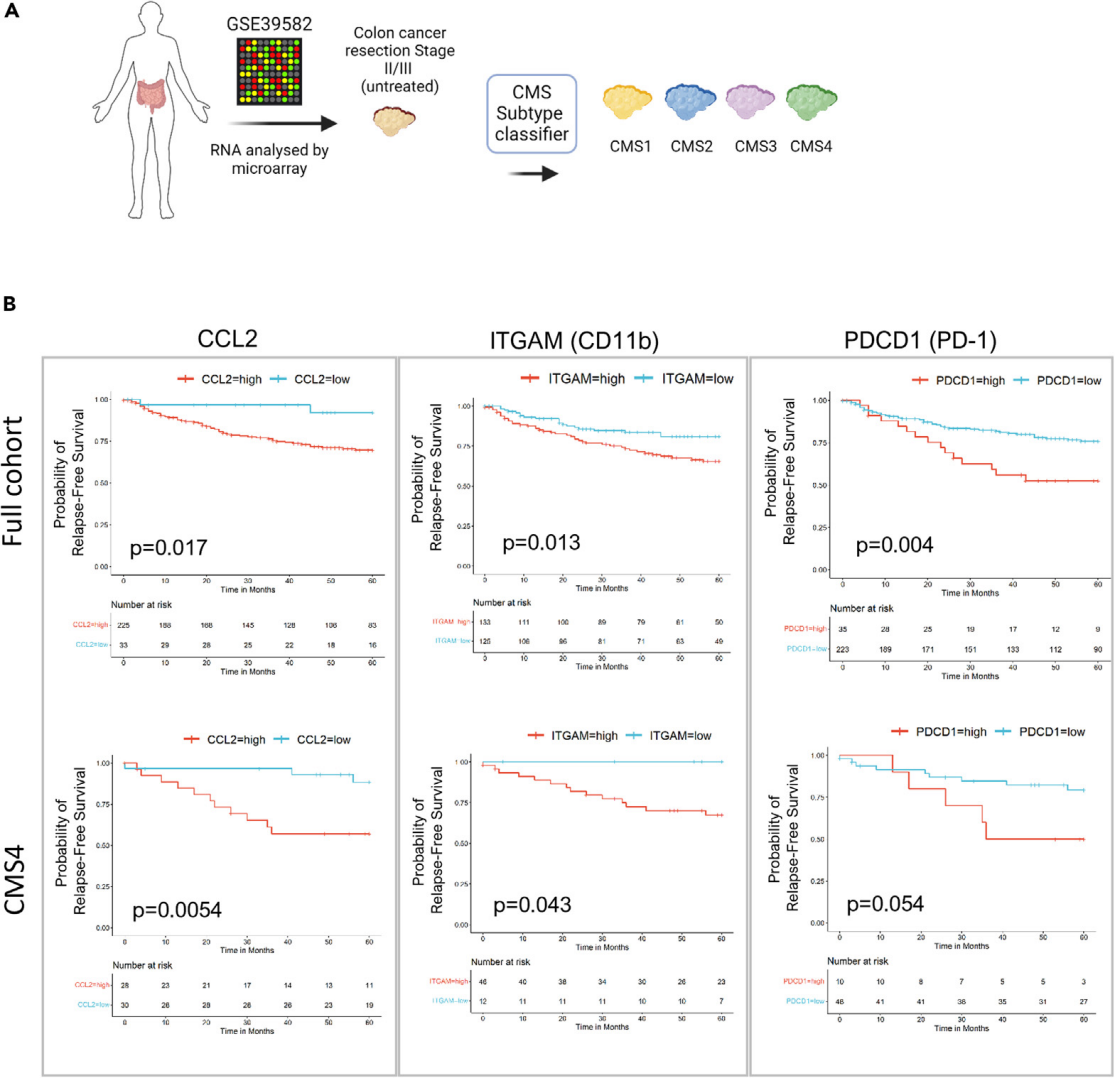
Association of CCL2, ITGAM and PDCD1 gene expression with relapse-free survival in untreated Stage II/III CRC tumors (A) Transcriptional profiles of stage II/III untreated colon cancer samples (GSE39582) were retrieved, and CMS classified (*n* = 258; CMS1 = 49, CMS2 = 75, CMS3 = 35, CMS4 = 58, unknown = 41). (B) Survival analysis for CCL2, ITGAM (CD11b) and PDCD1 (PD-1) in the entire cohort (*n* = 258) (top) or specifically in CMS4 samples (*n* = 58) (bottom). Kaplan-Meier survival curves showing log rank test *p* value.

Next, we assessed the prognostic relevance of genes involved in chemotaxis, macrophage infiltration and immunomodulation across both the entire stage II/III untreated CC cohort (*n* = 258), and specifically within CMS4 tumors (*n* = 58), (Figure 6A). Across the entire cohort, samples with a higher expression of CCL2 (Figure 6B, left), ITGAM (CD11b) (Figure 6B, middle) and PDCD1 (PD-1) (Figure 6B, right) had a significantly worse prognosis than those with lower expression. The same results were observed for CMS4 tumors, albeit with PD-1 approaching statistical significance (*p* value = 0.054) (Figure 6B, lower right). These results suggest the potential for assessing the functional impact of PD-1 signaling in macrophages which may have therapeutic implications in CMS4 tumors.

### Functional targeting of PD-1 restores activated macrophage phagocytosis in CMS4-like stromal cell co-cultures

**Figure 7:**
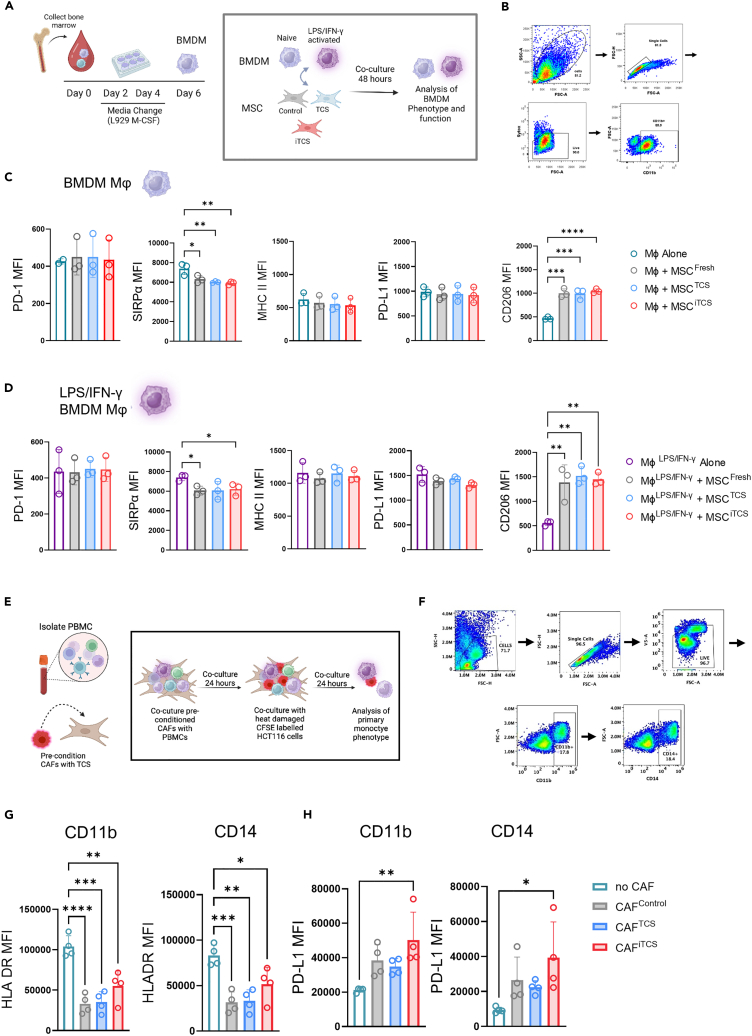
Tumor conditioned stromal cells regulate co-cultured macrophage phenotype (A) Experimental outline of Balb/c-derived BMDM isolation and subsequent culture and experimental setup utilized to co-culture BMDMs and MSCs. (B) Flow cytometry gating strategy used to analyze single, live macrophages following co-culture with MSCs. Flow cytometric analysis of PD-1, SIRPα, MHC-II, PD-L1 and CD206 expression on (C) naive or (D) IFN-γ+LPS activated BMDMs following co-culture with control and tumour-conditioned MSCs (*n* = 3). (E) Experimental outline of PBMC and CAF isolation and subsequent culture and experimental setup utilized to co-culture human PBMC derived mononuclear cells. (F) Flow cytometry gating strategy used to analyze single, live human mononuclear cells following co-culture with CAFs conditioned by human TCS. (G) Flow cytometric analysis of HLA-DR on co-cultured CD11b (left) CD14 (right) mononuclear cells. (H) Flow cytometric analysis of PD-L1 on co-cultured CD11b (left) CD14 (right) mononuclear cells (*n* = 4 independent CAF donors). Error bars, mean ± SD; ∗, *p* < 0.05; ∗∗, *p* < 0.01; ∗∗∗, *p* < 0.001; ∗∗∗∗, *p* < 0.0001 by one-way ANOVA and Tukey post hoc test.

To assess how stromal cells directly influence macrophage phenotype and function, naive or activated bone marrow-derived macrophages (BMDMs) were co-cultured with tumor conditioned-MSCs (Figure 7A). Immunomodulatory receptors and ligands were assessed on macrophages following co-culture with MSCs (Figure 7B). Expression of PD-1, MHC-II and PD-L1 were unchanged in both naive (Figure 7C) and activated macrophages (Figure 7D). Reduced expression of SIRP-α and induced expression of CD206 were observed in both naive and activated macrophages (Figures 7C and 7D). This data validated previous observations of an increased frequency of CD11b+ MHCII- CD206+ macrophages in CT26 stromal tumors (Figure 4G).

**Figure 8:**
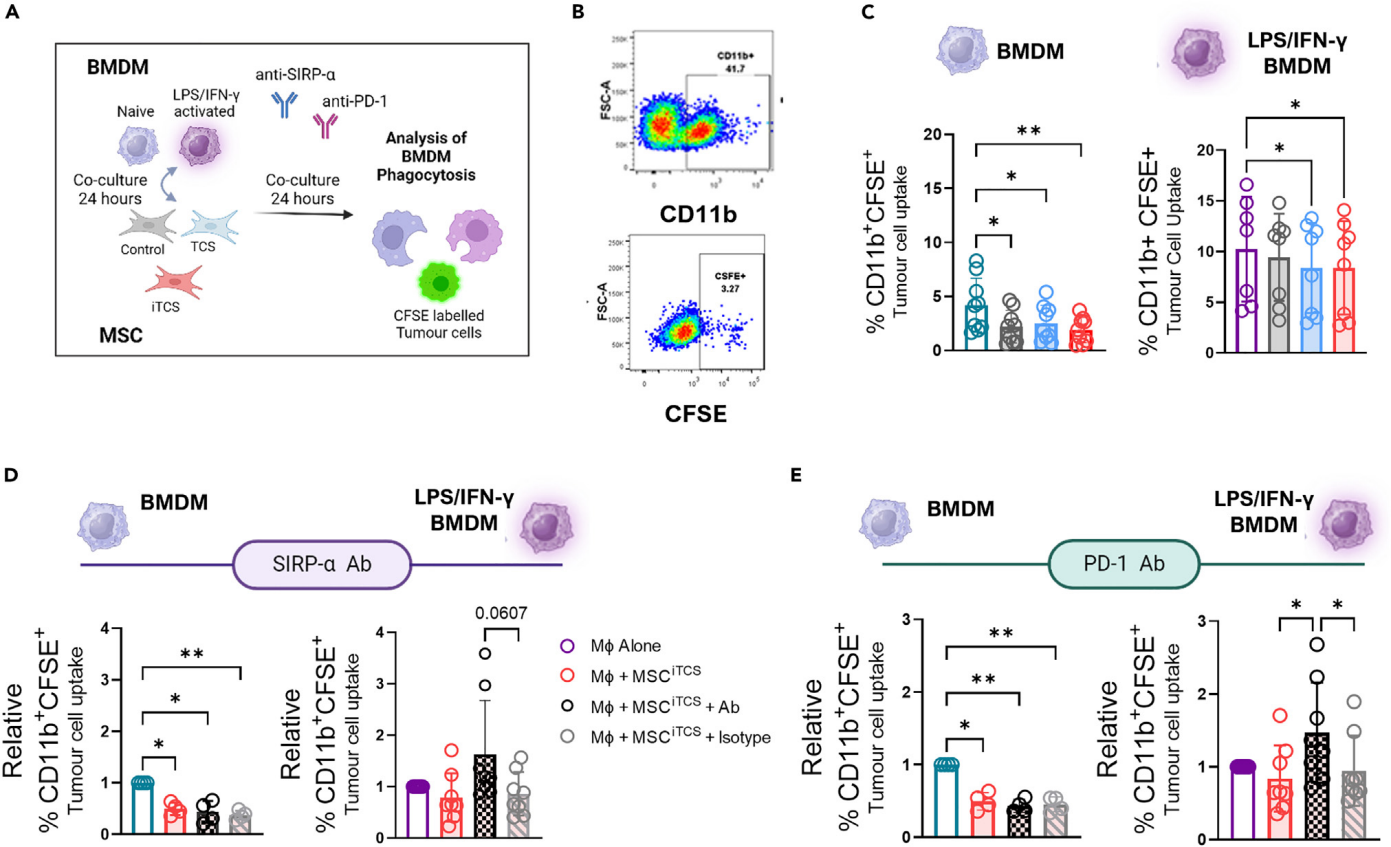
Stromal cells suppress macrophage phagocytic capacity by signaling through PD-1 (A) Experimental outline of Balb/c-derived BMDM isolation and subsequent culture and experimental setup utilized for co-culture BMDMs and MSCs. (B) Flow cytometry gating strategy used to analyze single, live macrophages which had taken up the fluorescently labeled (CFSE) cancer cells. (C) Tumor cell uptake was measured as the relative frequency of CD11b macrophages that were positive for CFSE following co-culture with conditioned or control stromal cells (relative to the macrophage alone group) (n = 7–9). (D) Data show relative frequency (relative to macrophages alone) of CFSE-expressing CD11b+ macrophages following internalization of CFSE-labelled cancer cells. Macrophages co-cultured with the iTCS MSCs treated with SIRPα blocking monoclonal antibody. Naive BMDM (Left) and LPS/IFN-γ BMDM (right) or (E) PD-1 blocking monoclonal antibody. Naive BMDM (Left) and LPS/IFN-γ BMDM (right) or relevant isotype control (n = 4–8). Note: for (D) and (A) (left) and (D) and (E) (right) the macrophage alone (Gray bar) and MSC^iTCS^ untreated group (Pink/red bar) show the same data. Error bars, mean ± SD; ∗, *p* < 0.05; ∗∗, *p* < 0.01; ∗∗∗, *p* < 0.001; ∗∗∗∗, *p* < 0.0001 by one-way ANOVA and Tukey post hoc test (C, D, and E).

We then assessed the effects of CAFs on human PBMC-derived monocytes *ex vivo* in the presence of tumor cells. Naive PBMC-derived monocytes were co-cultured with CAFs, control, or conditioned with TCS or iTCS and tumor cells (Figure 7E). Following co-culture, monocytes/macrophages were identified from live cells by CD14 and CD11b expression, respectively (Figure 7F). HLA-DR and PD-L1 expression were assessed as an indicator of immunosuppressive phenotypes. We observed a significant suppression of HLA-DR on CD11b and CD14 cells (Figure 7G, left) and a significant increase in PD-L1 expression on CD11b and CD14 cells, specifically following co-culture with iTCS treated CAFs (Figure 7G, right). Taken together, this data suggests that conditioned MSCs/CAFs alter macrophage phenotype and can induce PD-L1-expressing macrophages in human *ex vivo* co-cultures. We next investigated the effects of stromal cells on macrophage function. Informed by the transcriptional signatures in CMS4 tumors, we assessed macrophage phagocytosis of fluorescently labeled cancer cells in the presence or absence of conditioned mMSCs (Figure 8A). Uptake was quantified by flow cytometry (Figure 8B). Naive macrophages cultured with each of the MSC groups showed reduced cancer cell phagocytosis (Figure 8C, left). Similarly, although the baseline level of phagocytosis was higher, IFN-γ/LPS activated macrophages displayed significantly reduced phagocytosis when cultured with TCS and iTCS-treated MSCs (Figure 8C, right). To further assess the role of MSCs in modulating macrophage uptake and antigen processing, DQ Ovalbumin was added to macrophage-MSC co-cultures (Figure S7A). We found that MSCs, regardless of pre-treatment, reduced the ability of naive or activated macrophages to uptake and process antigens (Figures S7B–S7D). Together the data illustrate that stromal-macrophage interactions in the stromal compartment of the CRC TME alters macrophage phenotype and suppresses phagocytic function.

Both CD47 and PD-L1/PD-L2, upregulated on the iTCS-treated MSCs, are known to have immunomodulatory functions through binding their cognate receptors, SIRP-α and PD-1, respectively.^17^^,^^49^ We previously showed the impact of PD-L1 expression on T cell-mediated anti-tumor immunity.^19^ To determine the impact of MSC CD47 or PD-L1 expression on macrophage phagocytosis, we repeated the cancer cell phagocytosis assay (Figure 8D) with the addition of SIRP-α and PD-1 receptor-blocking antibodies. The addition of the SIRP-α antibody, PD-1 antibody or relevant isotype control antibody did not significantly alter baseline naive or activated macrophage phagocytosis (Figures S8A–S8D). In addition, the blocking antibodies did not alter macrophage phagocytosis when cultured alone or with TCS-treated MSCs, irrespective of macrophage activation status (Figures S8A–S8D). However, activated macrophages co-cultured with iTCS-treated MSCs (which expressed highest levels of CD47 and PD-L1) showed enhanced phagocytosis following blockade of SIRP-α (*p* = 0.0607) and significantly increased uptake upon PD-1 inhibition (Figures 8D and 8E). The effects of enhanced phagocytosis were exclusively observed in activated macrophage and not naive macrophage co-cultures (Figures 8D and 8E). These data validate the functional role of stromal cells in the inflammatory CRC TME and suggests that targeting the PD-1/PD-L1 and/or SIRP-α/CD47 axis in macrophages, in addition to an activation stimulus, may enhance anti-tumor effector functions, such as macrophage-mediated phagocytosis as well as T cell-mediated anti-tumor immunity.^19^

## Discussion

Here we studied the role of stromal cells in TNF-α-driven inflammatory TME of CRC and their ability to modulate macrophage phagocytic function via PD-1 signaling. Patients with CMS4 tumors have the lowest disease-free survival rates. In addition to the strong mesenchymal stromal signature, there is also a strong wound healing-like inflammatory signature in CMS4 tumors.^8^^,^^10^ We identified TNF-α signaling within CMS4 tumors as a prominent feature. TNF-α mediated inflammation in CRC has been reported previously, with increased serum concentration of TNF-α associated with disease stage and metastasis. Additionally, TNF-α levels inversely correlate with disease-free survival rates.^32^^,^^50^ TNF-α plays a role in CRC initiation, in particular in patients with inflammatory bowel disease (IBD).^51^ IBD patients who receive anti-TNF-α have lower levels of risk for CRC development.^52^ Inflammation has long been linked to the development of cancer but the mechanisms by which it contributes to the immunosuppressive TME are not fully understood. TNF-α altered the secretory phenotype of CRC cells to induce an immunomodulatory stromal cell phenotype. Our research supports the importance of inflammation in the context of stromal cell function in CRC.

The role of the mesenchymal stromal compartment in tumor progression and response to treatment has been previously highlighted.^53^^,^^54^ MSCs have been defined as precursors to CAFs^47^^,^^55^ and CAFs interact with cancer cells to promote tumor development through a variety of mechanisms.^56^^,^^57^ CAFs are recruited by the tumor from local fibroblasts or the bone marrow to induce a tumor-promoting environment.^58^^,^^59^ Recent studies have classified the fibroblast heterogeneity in the TME of breast cancer and melanoma through single-cell RNA sequencing.^60^^,^^61^^,^^62^ There are distinct sub-populations of CAFs in the TME, some with strong immunomodulatory properties while others display strong contractile or ECM deposition features.^60^ However, the assessment of stromal cell heterogeneity across the four CMSs has not yet been conducted. Further studies are required to define the localization of CAF subtypes in tumors and the functional CAF phenotypes that may enable identification of targets for immunotherapy.

In response to the inflammatory CRC secretome, MSCs/CAFs increase cytokine and chemokine production, and express potent immunomodulating molecules such as PD-L1 and CD47. In addition, MSCs significantly alter the secretory phenotype in a CMS4-like 3D CRC model, validated using both THP-1 and primary macrophages. These data support emerging research, highlighting that CAFs have crucial immunomodulatory and pro-tumor effects. MSCs/CAFs have the capacity to uptake, process, and present antigens, and induce the deletion of antigen-specific CD8^+^ T cells.^20^^,^^63^ CAFs in breast cancer with an immunosuppressive phenotype secrete CXCL12 that in turn recruits CD4^+^ T cells to the TME and are then polarized to regulatory T cells (Treg) by interacting with CAFs.^61^ As such, they may be an optimal immunotherapeutic target in stromal-rich tumors. Further research combining tumor cells, MSCs/CAFs, macrophages and T cells to assess subsequent T cell activation and infiltration using microfluidic devices could shed light on the potential impact of stromal-macrophage crosstalk on adaptive immunity *ex vivo*.

Despite the well-defined and distinct characteristics of the different CMS subtypes of CRC, our research displays some striking similarities between CMS1 and CMS4 tumors. This is of particular interest as patients with CMS1-like tumors are currently eligible for treatment with immune checkpoint inhibitors but the other ∼85% of CRC patients are not currently eligible.^64^ While CMS1 tumors are often referred to as the immune subtype, they are not the only subtype with an immune infiltrate. CMS4 tumors are enriched for immune cells from the monocytic and myeloid lineages.^13^ This suggests patients with CMS4 tumors may be good candidates for innate immunity-targeting immunotherapeutic treatment. Preliminary data from clinical trials (NCT04126733, NCT03406871, NCT02870920, NCT05608044) treating microsatellite stable CRC patients with a combination of anti-PD-1 antibodies or PD-1-targeting drugs with either anti-CTLA4 antibody or kinase inhibitors (regorafenib) have suggested beneficial response rates between 7% and 36%.^65^^,^^66^^,^^67^ However, these studies did not evaluate the CMS of CRC tumors. It would be of interest to assess whether CMS4 subtype may have higher response rate based on their inflammatory stromal dense microenvironment.

CMS stratification has been shown to identify molecular signatures that dictate treatment response rates and TME signatures, however, has not yet been adapted in the clinical setting. CMS classification was initially based on a transcriptional profile derived from bulk analysis of tumor tissues,^9^ which is costly and restricted in terms of clinical application. However, Sirinukunwattana et al., 2021^11^ have employed deep learning to generate a CMS classifier from CRC H&E tissue sections. This development enables the CMS classification of CRC in a clinical setting with minimal extra cost, time, reagents, or expertise. This approach has the potential to make CMS stratification of CRC patients clinically possible in a cost-effective way, which may ultimately lead to guided treatment regimens for patients. The potential to classify tumors at diagnosis may have implications for identifying potential therapies or therapeutic combinations.

Our data have demonstrated that there is a complex mix of pro-tumor and anti-tumor macrophages in CMS4 tumors. Knowledge of targeted therapies to take advantage of the different macrophage phenotypes is critical for harnessing their anti-tumor potential. In a recent study Corry and colleagues have shown that the inflammatory stimulus poly (I:C) can activate innate immune cells and expose a therapeutic vulnerability in stromal-rich tumors.^68^ This may be of particular interest as targeting PD-1/SIRP-α had a more significant impact on pro-inflammatory activated macrophages. Whether this *in situ* activation profile of the macrophage is sufficient to allow the response to the PD-1 blocking antibody or if an additional activation is required is unknown. However, it has been shown that poly(I:C) increases macrophage antigen processing and reduces metastatic tumor burden in a CMS4-like liver metastasis animal model.^68^ It has recently been shown that immunotherapy-activated T cells can recruit and skew late-stage pro-inflammatory macrophages that can enhance anti-tumor efficacy.^31^ Taken together, these data support the combination of a PD-1 blocking antibody with a pro-inflammatory activation signal, such as poly(I:C), which may initiate an adaptive and innate immune-mediated anti-tumor response in CMS4 patients.

Our findings suggest the potential impact of TNF-α-induced inflammation in stromal-rich CRC. We showed effects of inflammatory tumor-conditioned MSCs on suppressing macrophage phagocytosis through PD-1 signaling. We found that CMS1 and CMS4 tumors have similar TNF-α-mediated and macrophage-related signaling, although CMS4 tumors with enhanced stromal infiltration demonstrate enhanced immunosuppression. This data may suggest the re-stratification of CRC patients by CMS subtype in the clinic may reveal a subset of patients with microsatellite stable, CMS4-like tumors that may respond positively to macrophage- or stromal-targeting immunotherapies.

### Limitations of the study

One limitation of this study relates to the factors that dictate stromal-mediated effects on functional macrophage phenotypes *in vivo*. It is likely that multiple factors contribute to stromal effects on macrophage infiltration and polarization. CRISPR screening of stromal cells could reveal mechanisms of stromal-mediated macrophage polarization. Additionally, changes in stromal-cell macrophage interactions are likely to be dynamic so time point analysis of macrophage functional phenotypes in GEMM mouse models of CRC using spatial imaging and scRNA sequencing may shed further light on targetable stromal cell-macrophage interactions to enhance ant-tumor immunity in stromal rich CRC TMEs.

## Resource availability

### Lead contact

Further information and requests for resources and reagents should be directed to and will be fulfilled where possible by the lead contact, A.R. (aideen.ryan@universityofgalway.ie).

### Materials availability

This study did not generate any new unique reagents.

### Data and code availability

• The RNA sequencing data presented in this study have been deposited in NCBI’s Gene Expression Omnibus and are accessible through GEO Series accession number GSE229054.
• This paper does not report original code.
• Any additional information required to reanalyze the data reported in this paper is available from the lead contact upon reasonable request.

## Acknowledgments

The authors acknowledge the facilities and scientific and technical assistance of the Anatomy Imaging and Microscopy Facility at the 10.13039/501100001634University of Galway. All flow cytometry experiments were performed in the University of Galway Flow Cytometry Core Facility, which is supported by funds from 10.13039/501100001634University of Galway, 10.13039/501100001602Science Foundation Ireland, the Irish Government’s Programme for Research in Third Level Institutions, Cycle 5 and the 10.13039/501100008530European Regional Development Fund. Technical and consultative support for flow cytometry experiments was provided by Dr Shirley Hanley of the University of Galway Flow Cytometry Core Facility. Technical support was provided in the Lambe Institute for Translational Research at the University of Galway by Coralie Mureau and Catherine Loughrey. Graphics and animations created with BioRender, Microsoft PowerPoint and Servier Medical Art. Servier Medical Art by Servier is licensed under a Creative Commons Attribution 3.0 Unported License (https://creativecommons.org/licenses/by/3.0/). The authors wish to thank the Bio-Resources Unit (BRU) technical, veterinary, and administrative staff in the 10.13039/501100001634University of Galway for facilitating *in vivo* studies and for their ongoing assistance, advice, and support in animal procedures, husbandry, care, and welfare. The authors wish to acknowledge Prof Frances Balkwill for supporting a research sabbatical to 10.13039/501100019154Barts Cancer Institute which facilitated this study. Finally, we wish to acknowledge and thank the patients who, following informed consent, provided samples for this study, for which we are extremely grateful.

This study was supported primarily by a 10.13039/501100001602Science Foundation Ireland Starting Investigator Award (15/SIRG/3456) and an SFI Frontiers for the Future Award (19/FFP/6446) and 10.13039/501100019905Galway University Foundation funding to A.E.R. A.E.R and D.L were supported in part by the Organ-on-a-Chip Technologies Network, which is funded by UKRI via the Technologies Touching Life Scheme (Grant reference: MR/R02569X/1). E.R. was supported by an SFI CDT Lifetime Scholarship (co funded by UK Research and Innovation (UKRI) -Engineering and Physical Sciences Research Council (EPSRC) 18/EPSRC-CDT/3583; EP/S02347X/1; 13/RC/2073_P2). KT funded by 10.13039/100014989Chan Zuckerberg Initiative (CZI) grant DAF2021-225429 and grant DOI https://doi.org/10.37921/723688hijigu. S.M.C and P.D. are funded by 10.13039/501100000289Cancer Research UK early detection project (A29834).

## Author contributions

N.A. L. led the study, contributed to study design and performed the majority of experiments. E.R. and S.M.C. performed majority of experiments and associated data analysis for the revised manuscript. A.O. and S.H. performed immunohistochemical analysis of human CRC tumors. H.E. and O.T. performed *in vivo* experiments and data analysis. G.O. performed RNA sequencing and cytokine analysis. M.S., A.C., L.J.E., and A.M.H. planned and performed surgical resections and biopsies and pathological assessment of the samples. K.T and E.M.. performed the SEM and TEM image acquisition and analysis. D.L. conceived the 3D model study design and data interpretation. N.K. contributed to manuscript writing and data analysis. S.M.C., K.R., and P.D performed analysis on human CRC datasets and contributed to the interpretation of data and manuscript writing. T.R. contributed to study design and manuscript writing. A.E.R. conceived the study and study design, experimental planning, data interpretation and manuscript writing

## Declaration of interests

The authors declare no conflicts of interest.

## STAR★Methods

### Key resources table

**Table:** 

REAGENT or RESOURCE	SOURCE	IDENTIFIER
**Antibodies**		

Anti human CD73-FITC (Clone AD2)	Biolegend	RRID:AB_2561808; Cat No: 344016
Anti-human CD45 -BD Horizon V500 (Clone HI30)	BD Biosciences	RRID:AB_1937324; Cat No: 560777
Anti-human CD105 -APC (Clone 43A3)	Biolegend	RRID:AB_755960; Cat No: 323207
Anti-human PDGFR-a -PE/Cy7 (Clone 16A1)	Biolegend	RRID:AB_755996; Cat No: 323502
Anti-human PDGFR-b -APC (Clone 18A2)	Biolegend	RRID:AB_2162787; Cat No: 323608
Anti-human Podoplanin -APC/Cy7 (Clone NC-08)	Biolegend	RRID:AB_2750290; Cat No: 337029
Anti-human CD47 -PE/Cy7 (Clone CC2C6)	BioLegend	RRID:AB_2565514; Cat: 323114
Anti-human CD90 -BV510 (Clone 5E10)	BioLegend	RRID:AB_2562199; Cat: 328125
Anti-human EpCAM -FITC (Clone 9C4)	BioLegend	RRID:AB_756078; Cat: 324204
Anti-human FAP -APC (Clone 427819)	R and D Systems	RRID:AB_2884010; Cat: FAB3715A
Anti-human PDGFRα -PE/Cy7 (Clone 16A1)	BioLegend	RRID:AB_2565596; Cat: 323507
Anti-human CD47 -PE/Cy7 (Clone CC2C6)	BioLegend	RRID:AB_2565514; Cat: 323114
Anti-human PD-L1 -PE (Clone 29E.2A3)	BioLegend	RRID:AB_940368; Cat: 329706
Anti-human PD-L2 -APC/Cy7 (Clone MIH18)	BioLegend	RRID:AB_2783234; Cat: 345516
Anti-mouse/human CD11b -PerCP (Clone M1/70)	BioLegend	RRID:AB_2129374; Cat: 101230
Anti-mouse CD206 -APC(Clone C068C2)	BioLegend	RRID:AB_10900231; Cat: 141708
Anti-mouse CD47 -FITC (Clone miap301)	BioLegend	RRID:AB_1134140; Cat: 127504
Anti-mouse MHC Class II -FITC (Clone M5/114.15.2)	Tonbo Biosciences	RRID:AB_2621715; Cat: 35-5321
Anti-mouse PD-1 -BV421 (Clone 29F.1A12)	BioLegend	RRID:AB_2561447; Cat: 135218
Anti-mouse PD-L1 -PE (Clone 10F.9G2)	BioLegend	RRID:AB_2073556; Cat: 124308
Anti mouse SIRPα -APC/Cy7 (Clone P84)	BioLegend	RRID:AB_2629558; Cat: 144018
Ultra-LEAF™ anti-mouse PD-1 blocking antibody (Clone 29F.1A12)	BioLegend	RRID:AB_2783089; Cat: 135246
Isotype for PD-1 blocking antibody (Clone RTK2758)	BioLegend	RRID:AB_11147167; Cat: 400544
Ultra-LEAF™ anti-mouse SIRPα (clone P84)	BioLegend	RRID:AB_2832518; Cat: 144037
Anti-human CD68 (Clone PG-M1)	Dako	RRID:AB_2074844; Cat No: M0876
Anti-human CD163 (Clone D6U1J)	Cell Signaling Technology	RRID:AB_2800204; Cat: 93498
Anti-mouse CD45-BV510 (Clone 30-F11)	Biolegend	RRID:AB_2563061; Cat No: 103138
Anti-mouse/human CD11b-BV421 (Clone M1/70)	Biolegend	RRID:AB_11203704; Cat No: 101236
Anti-mouse MHC-II-PerCP 5.5 (Clone M5/114.15.2)	TONBO biosciences	Cat No: 65-5321-U100
Anti-mouse CD206-PE-Dazzle (Clone C068C2)	Biolegend	RRID:AB_2565932; Cat No: 141732

**Biological samples**		

Colorectal cancer patient-derived tumor sections	Galway University Hospital	N/A
Human bone marrow (healthy donors)	Galway University Hospital	N/A
Human cancer associated fibroblasts	Galway University Hospital	N/A
Human peripheral blood mononuclear cells	Galway University Hospital	N/A

**Chemicals, peptides, and recombinant proteins**		

DMEM medium (high glucose)	Sigma Aldrich	Cat No: D5796
McCoys 5A medium	Sigma by Life Science	Cat No: M8403
RPMI-1640 medium	Gibco	Cat No: A10492-01
MEM-α medium	Gibco	Cat No: 32561-029
Human FGF-basic	PeproTech	Cat No: 100-18B
TNF-alpha	PeproTech	Cat No: 300-01A
Recombinant Murine IFN-γ	Peprotech	Cat No: 315-05
Recombinant Murine TNF-alpha	Peprotech	Cat No: 315-01A
LPS	Sigma Aldrich	Cat No: L4391-1MG
DAB	Abcam	Cat No: ab64238
DQ-Ovalbumin	Thermo Fisher Scientific	Cat No: D12053
GelMA	Prepared as per Loessner et al.^38^	
Photo-initiator 2-Hydroxy-4′- (2-hydroxyethoxy)-2-methylpropiophenone	Sigma-Aldrich	Cat No: 410896-10G
TruStain FcX™ PLUS FC block (Clone: S17011E)	Biolegend	Cat No: 156604
Ficoll®-Paque Premium	Sigma Aldrich	Cat No: GE17-5442-02
ACK Lysis Buffer	Gibco	Cat No: A10492-01
Glutaraldehyde	Sigma Aldrich	Cat No: 111-30-8
Paraformaldehyde	Sigma Aldrich	Cat No: 30525-89-4
Sodium cacodylate trihydrate	Sigma Aldrich	Cat No: 6131-99-3
Ethanol	Sigma Aldrich	Cat No: 51976
Hexamethyldisilazane	Sigma Aldrich	Cat No: 999-97-3
Osmium tetroxide	Sigma Aldrich	Cat No: 20816-12-0
Agar Low-Viscosity Resin	Agar Scientific	Cat No: AGR1078
Toluidine blue	Sigma Aldrich	Cat No: 6586-04-5
Dulbecco’s phosphate buffered saline (PBS)	Sigma Aldrich	Cat No: D8537
Sodium Azide	Sigma Aldrich	Cat No: S2002
Sodium pyruvate	Sigma Aldrich	Cat No: S8636
HEPES solution	Sigma Aldrich	Cat No: H0887
L-glutamine	Gibco	Cat No: 25030024
Fetal Bovine Serum	Sigma by Life Science	Cat No: F7524
Penicillin/streptomycin	Gibco	Cat No: 15140-122
β-mercaptoethanol	Gibco	Cat No: 31350-010
Sodium Chloride (NaCl)	Sigma Aldrich	Cat No: S7653
Nuclease free water	Promega	Cat No:P119E
0.5M EDTA	Sigma Aldrich	Cat No: 03690
RNAseA (1.4 U/ml)	Thermofisher scientific	Cat No: 12091021
Human Serum	Sigma Aldrich	Cat No: H4522
Collagenase from Clostridium histolyticum	Sigma Aldrich	Cat No: C2674

**Critical commercial assays**		

CellTrace CSFE, Cell Proliferation Kit, for flow cytometry	ThermoFisher Scientific	Cat No: C34554
SYTOX Blue Dead Cell Stain	ThermoFisher Scientific	Cat No: S34857
SYTOX AADvanced Dead Cell Stain Kit	ThermoFisher Scientific	Cat No: S10349
Tumor Dissociation Kit, human	Miltenyi Biotec	Cat No: 130-095-929
Tumor Dissociation Kit, mouse	Miltenyi Biotec	Cat No: 130-096-730
LIVE/DEAD™ Viability/Cytotoxicity Kit	(Invitrogen, Thermo Fisher Scientific)	Cat No: L3224
The Proteome Profiler Human Cytokine Array Kit	R&D systems	Cat No: ARY005B
Zombie Violet	Biolegend	Cat No: 423114
Human Monocyte Isolation kit	Miltenyi Biotec	Cat No: 130-096-537
Human Tumor Dissociation Kit	Miltenyi Biotec	Cat No: 130-095-929
AlamarBlue™ cell viability assay	Invitrogen	Cat No: DAL1100
CyQUANT® Cell Proliferation Assay Kit	Invitrogen	Cat No: C7026

**Experimental models: Cell lines**		

CT26	American Type Culture Collection (ATCC)	RRID:CVCL_7256 Cat No: CRL-2638
HCT116	American Type Culture Collection (ATCC)	RRID: CVCL_0291 Cat No:CCL-247
HT29	American Type Culture Collection (ATCC)	RRID:CVCL_0320 Cat No: HTB-38
THP1	American Type Culture Collection (ATCC)	RRID:CVCL_0006 Cat No: TIB: 202

**Experimental models: Organisms/strains**		

Balb/c	Envigo Laboratories	Balb/cAnNCrl

**Software and algorithms**		

BioRender	Biorender.com	RRID:SCR_018361
FlowJo Version 10	Tree Star Inc.	RRID:SCR_008520
GraphPad Prisim® Version 9	GraphPad	RRID:SCR_002798
QuPath	Qupath.github.io	RRID:SCR_018257
GenePattern	Genepattern.org	RRID:SCR_003201
Bioplex software	Bio-Rad	RRID:SCR_014330
ComplexHeatmap (2.6.2)	Bioconductor	Lynch et al.^69^; RRID:SCR_017270
Circlize (0.4.15)		Hänzelmann et al.^70^; RRID:SCR_002141
ggplot2 (3.4.1)		Subramanian et al.^71^; RRID:SCR_014601
ggbeeswarm (0.7.1)		Liberzon et al.^72^
ggpubr (0.6.0)		Gu et al.^73^
xCell (1.1.0)		Gu et al.^74^
MCPcounter (1.2.0)		Wickham^75^
ConfoundR	Confoundr.qub.ac.uk	

### Experimental model and study participant details

#### Mice

Balb/c mice (aged 8–14 weeks, female) were purchased from Envigo Laboratories (Oxon, UK). Ethical approval for this research was granted by the Animal Care Research Ethics Committee (ACREC) committee of University of Galway (ACREC-17-Dec-04). Experiments were conducted under individual and project authorisation licences from the Health Products Regulatory Authority (HPRA) of Ireland (AE19125/P077). Female mice were acclimatised for at least 7 days prior to tumor induction, MSC or BMDM isolation. All mice were housed in individually ventilated cages under specific pathogen-free facility, fed ad lib on a standard chow diet, and cared for under the standing operating procedures in the BRU Animal Facility at the University of Galway, Ireland.

#### Tumor cell lines

Balb/c-derived CT26 colon carcinoma cells were purchased from the American Type Culture Collection (ATCC, VA, USA) and cultured in DMEM media (Thermo Fisher Scientific) supplemented with 10% FBS (Sigma-Aldrich) and 1% penicillin/streptomycin (Thermo Fisher Scientific). HT29 and HCT116 human colon carcinoma cells were also purchased from ATCC and cultured in McCoys 5A medium (Sigma-Aldrich) supplemented with 10% FBS (Sigma-Aldrich), L-glutamine and 1% penicillin/streptomycin (Thermo Fisher Scientific). THP-1 cells were subcultured in RPMI-1640 media (Thermo Fisher Scientific) supplemented with 10% FBS, 1% glutamine and 1% penicillin/streptomycin (Thermofisher Scientific). Cell line master stocks were authenticated by ATCC, confirmed mycoplasma negative, expanded, frozen and used within 15 passages.

#### Human samples

Human colorectal cancer samples, including formalin-fixed paraffin-embedded samples (FFPE), peripheral blood samples and fresh resection samples were obtained from the Surgical and Pathology Departments of Galway University Hospital, pseudonymised and processed for research purposes under ethical approval (Clinical Research Ethics Committee, Ref: C.A. 2074). Written informed explicit consent was obtained from all patients prior to sampling.

#### Isolation of bone marrow-derived mesenchymal stromal cells

Murine bone marrow-derived MSC (mMSC) were isolated and cultured as previously described.^19^ Briefly, mice were euthanized by CO_2_ inhalation and the femur and tibia were removed, cleaned of connective tissue, and placed in sterile dPBS. The bones were flushed with 30G needles, and cell suspensions were filtered through 70μm mesh filters and centrifuged at 400 × g for 5 min. Red blood cells were removed using ACK lysis buffer (Thermo Fisher Scientific, Dublin, Ireland). The cells were cultured in complete media consisting of MEM-α (Thermo Fisher Scientific, Dublin, Ireland) supplemented with 10% heat-inactivated fetal bovine serum (HI-FBS) (Sigma-Aldrich, Wicklow, Ireland), and 1% penicillin/streptomycin (ThermoFisher Scientific). Non-adherent cells were removed 24 h later and cells were refreshed with fresh media. This process was repeated until cells reached confluency.

Human bone marrow-derived MSC (hMSCs) were isolated as described previously^19^ and conducted at Galway University Hospital under an ethically approved protocol (NUIG Research Ethics Committee, ref. 08/May/14) and under written consent from the volunteers (2 male and 1 female). Briefly, bone marrow cell suspensions were layered onto a Ficoll density gradient. The nucleated cell fraction was collected, washed, and resuspended in hMSC culture medium. 24 h later, non-adherent cells were removed, and fresh medium was added. Individual colonies of fibroblast-like cells were allowed to expand and approach confluence prior to passage. hMSC culture medium contains MEM-α (Thermo Fisher Scientific) supplemented with 10% HI-FBS (Sigma-Aldrich), 1% penicillin/streptomycin (Thermo Fisher Scientific) and 1 ng/mL fibroblast growth factor 2 (FGF2; Peprotech, London, UK). MSCs were characterized according to the criteria set out by the International Society for Cellular Therapy (ISCT). Cell surface characterization and tri-lineage differentiation was performed as per.^76^

#### Isolation of colorectal cancer-associated fibroblasts

Colorectal patient-derived cancer-associated fibroblasts (CAFs) or normal-associated fibroblasts (NAFs) ((2 male; 2 female) were isolated from fresh tumor resections at University Hospital Galway under an ethically approved protocol (Clinical Research Ethics Committee, Ref: C.A. 2074) as outlined in.^22^ Following pathological assessment, biopsies from center tumor and adjacent normal mucosal tissue were removed and single-cell suspensions were prepared aseptically using a human Tumor Dissociation Kit (Miltenyi Biotech) according to the manufacturer’s protocol with some modifications. The cell suspensions were cultured in complete medium containing RPMI-1640 (Thermo Fisher Scientific) supplemented with 10% FBS, 1% sodium pyruvate, 1% HEPES solution, 1% L-glutamine, 1% penicillin/streptomycin, 0.1% β-mercaptoethanol (all Sigma-Aldrich) and 1 ng/mL FGF2 (Peprotech). Cells were then seeded in 6-well plates (Sarstedt) until stromal cell colony establishment was observed. CAFs and NAFs were characterised by expression of CD73-FITC, CD45-BV500, CD105-APC, PDGFR-a-PE/Cy7, PDGFR-b-APC, Podoplanin-APC/Cy7 and HLA-DR-PE/Cy7.

#### Isolation of Peripheral Blood Mononuclear Cells (PBMCs)

PBMC isolation research protocols were approved by the Clinical Research Ethics Committee at University Hospital Galway (Ref C.A. 2534). All blood donors provided written informed consent for this study. Peripheral blood mononuclear cells (PBMCs) were isolated from fresh blood samples taken through the hemochromatosis clinic at University Hospital Galway. Briefly, blood was separated into its components via a ficoll spin at 2000 rpm for 20 min (1 acceleration/0 deceleration).^22^ The buffy coat was harvested and pelleted by spinning at 400 g for 5 min. The pellet was washed twice with sterile Dulbecco’s phosphate buffered saline (PBS) (Sigma Aldrich), after which the PBMC pellet was resuspended in media.

#### Bone marrow derived macrophage isolation and culture

Balb/c bone marrow-derived macrophages (BMDMs) were isolated and cultured as previously described.^69^ Single cell suspension from bone marrow were prepared as described above. Briefly, mice were euthanized by CO_2_ inhalation and the femurs and tibias were removed, cleaned of connective tissue, and placed in sterile DPBS. The bones were flushed with 30G needles, clumps were filtered through 70μm mesh filters and centrifuged at 400 × g for 5 min. Red blood cells were removed using ACK lysis buffer (Thermo Fisher Scientific). Bone marrow cells were cultured at a density of 4.5 × 10^6^ per well of a low adherent six-well plate (Corning) in macrophage medium for 6 days, with media changed every two days. Macrophage media consisted of 65% complete medium and 35% L929 conditioned medium. Complete medium contained RPMI 1640 supplemented with 10% heat-inactivated FBS, 1% sodium pyruvate, 1% non-essential amino acid (0.1 mmol/L), 1% L-glutamine (2 mmol/L), 1% penicillin (100 U/mL)/streptomycin (100 μg/mL), and 0.01% β-mercaptoethanol (55 μmol/L) (all from Sigma-Aldrich). L-929 conditioned medium containing macrophage colony stimulating factor (M-CSF), was collected from L929 cells. BMDMs were harvested after 6 days using 0.25% trypsin-EDTA (Thermo Fisher Scientific) and incubated for 6–10 min at 37°C. If required, BMDMs were stimulated with 100 ng/mL IFN-γ (Peprotech) for 16 h followed by stimulation with 10 ng/mL LPS (Sigma-Aldrich) for 4 h.

### Method details

#### Generation of tumor cell secretome and stromal cell conditioning

To generate tumor cell secretome (TCS), CT26, HT29 or HCT116 cells were seeded in T175 flasks at a density of 1x10^6^, 2 x10^6^, or 1.5x10^6^ cells in 25 mL of the relevant media, respectively. Cells were grown at 37°C for a total of 72 h, whereupon the conditioned medium was collected and spun at 1000 × g. The pellet was discarded, and the conditioned medium was aliquoted and stored at −80°C. To generate inflammatory TCS (iTCS), cells were seeded at the same density with the addition of 100 ng/mL human or mouse TNF-α (Peprotech) 24 h prior to secretome harvesting.

For human stromal cells tumor conditioning studies, MSCs and CAFs were seeded at a density of 6 × 10^4^ or 7.5 × 10^4^, respectively, per well of a 6-well plate in 2 mL of complete culture medium. 24 h later, medium was removed and replaced with 40% fresh medium +60% TCS or iTCS and cultured for another 48 or 72 h. Additionally, TCS or iTCS heated to 95°C for 20 min to destroy active factors in the media or fresh media with 0.5 ng/mL TNF-α (Peprotech) were used where indicated. Conditioned stromal cells were collected, washed twice in DPBS (Sigma Aldrich). The cells were then stained for cell surface expression of target markers (see key resources table) and analyzed by flow cytometry or used in co-culture assays. Additionally, secretome from conditioned cells was collected, spun at 1000 × g to remove cell debris and stored at −80°C for later use.

#### Transcriptional analysis

The subset of the stage II/III untreated colon cancer (CC) microarray dataset of the stage II/III untreated colon cancer, GSE39582) [26] (*n* = 258; CMS1 = 49, CMS2 = 75, CMS3 = 35, CMS4 = 58, unknown = 41) was downloaded and normalized as previously described.^68^ Samples were classified into the consensus molecular subtypes (CMS) using the CMS classifier package (1.0.0) by the random forest method.^8^ Single sample gene set enrichment analysis (ssGSEA) scores were generated using the GSVA package (1.38.2).^70^^,^^71^ The genesets used were retrieved from the Molecular Signatures Database (MSigDB)^71^^,^^72^ and imported into the R package (4.0.5). The scores were scaled using the scale function (default settings) in R with the gene sets as columns. The two packages used to generate heatmaps were ComplexHeatmap (2.6.2)^73^ and circlize (0.4.15).^74^ The boxplots were created using ggplot2 (3.4.1),^75^ ggbeeswarm (0.7.1)^77^ and statistically annotated by ggpubr (0.6.0).^78^ The statistical test used was Wilcoxon Rank-Sum test with CMS4 as the reference group. The microenvironment scores were generated using xCell (1.1.0)^79^ and MCPcounter (1.2.0).^80^ The ConfoundR web was used to generate and download figures throughout [36]. Pearson’s correlation was calculated using stats package (4.0.5).^38^ Survival analysis was carried out using the survminer (0.4.9), KMsurv (0.1–5) and survival (3.5–5) packages. For each gene, the surv_cutpoint() function was used to find the cutpoint which maximised the difference in relapse free survival (5 years). The identified cutpoint was then used to dichotomise the samples into high and low groups for survival analysis. Kaplan-Meier survival curves were estimated using the survfit() function and visualised using ggsurvplot().^75^

#### RNA sequencing analysis

RNA sequencing of tumour-conditioned murine MSCs was performed exactly as detailed in.^22^ Gene set enrichment analysis (GSEA) was performed on differentially expressed genes (filtered based of fold change cutoff 1.5; *p*-value ≤0.05) using GenePattern (https://www.genepattern.org/).

#### Cytokine quantification

Tumor cell secretome was analyzed using the Bio-Rad Bioplex 200 (Bio-Rad – Fannin Healthcare) according to the manufacturer’s instructions. Cytokine concentrations were calculated and plotted as pg/mL based on the standard curve generated by the Bioplex software (Bio-Rad).

#### Multicellular 3D model of CMS4 tumors

GelMA hydrogels were prepared as reported in.^21^^,^^42^ Cells were re-suspended in 5% (w/v) GelMA solution at the following concentrations: 1.75 × 10^5^ HCT116 cells/mL, 8.75 × 10^4^ THP-1 cells/mL or primary human monocytes, 8.75 × 10^4^ MSCs/mL. 3D cell cultures with THP-1 cells were grown in 50% HCT116 medium and 50% hMSC medium. 3D cell cultures with primary monocytes were grown in 50% hMSC medium and 50% monocyte medium. The media was changed on days 1 and 8. TNF-α (Peprotech) was added to relevant wells at 100 ng/ml 3D cell cultures were assessed at day 10.

#### Isolation of primary monocytes

In order to assess primary monocytes role in a co-culture model, PBMCs were isolated from healthy patient blood, by layering whole blood on Ficoll-Paque Premium (Sigma Aldrich) and spinning at 2000 RCF for 30 min. A pan human monocyte isolation kit (Miltenyi Biotec) was used to isolate CD14 positive monocytes from 5 × 10^7^ PBMCs. Monocytes were plated in a 6 well plate for 24 h prior to co-culture in multicellular 3D CRC model. Medium for monocyte growth consisted of RPMI media with 10% human serum (Sigma Aldrich), 1% L-glutamine and 1% penicillin/streptomycin.

#### Analysis of 3D cell cultures

Cell metabolic activity was determined by AlamarBlue assays as previously reported [38]. Briefly, media was removed from wells containing gels and 500 μL of media containing 10% AlamarBlue solution (Invitrogen) was added to each well. Plates were incubated at 37°C for 8 h. Following incubation, measurements were taken by placing 100 μL of AlamarBlue solution per 3 wells of a flat bottom 96 well plate. Absorption was measured at 544 nm and 590 nm. Cell viability was assessed using a LIVE/DEAD Viability/Cytotoxicity Kit (Invitrogen, Thermo Fisher Scientific) as described previously.^81^ Briefly, a live/dead mix was prepared by Calcein AM at a concentration of 1/1000 and propidium iodide at a concentration of 1/2000 in dPBS. Gels were transferred to a 24-well plate using a curved spatula. Gels were washed for 15 min using dPBS at 37°C on a shaker. DPBS was then removed and 300 μL of the live/dead mix was added to each well. Gels were incubated for 30 min at 37°C. Gels were then transferred to a glass bottom Petri dish and covered with dPBS for imaging. Samples were imaged using the FLUOVIEW FV3000 confocal laser scanning microscope (Olympus). Cell viability was also assessed following dissociation of the hydrogels and spheroids by flow cytometry (detailed below).

Spheroid morphology was assessed by scanning electron microscopy (SEM) and cellular morphology within the spheroid by transmission electron microscopy (TEM) as outlined in [19] and described in detail below. The Proteome Profiler Human Cytokine Array Kit (ARY005B, R&D systems, Minnesota, United States) was used to assess the secretion of cytokines. The 3D culture conditioned medium was collected and analyzed according to the manufacturer’s recommendations and as previously described.^21^ Signal density was analyzed using ImageJ.

Additionally, for primary monocyte 3D co-cultures, proliferation of cells was assessed using the CyQUANT Cell Proliferation Assay Kit (Invitrogen, Ref no. C7026). Briefly, gels were frozen at −80°C with 1 gel placed per Eppendorf. Gels were dissociated using human tumor dissociation kit (Miltenyi) for 1.5 h at 37°C. After 1.5 h, gels were centrifuged at 1000 g for 30 s. RNAse solution for each sample was prepared by combining 105.5 μL 360 nM NaCl (Sigma Aldrich), 4.5 mL Nuclease free water (Promega), 15 μL of 1× Cell lysis buffer, 20 μL of 0.5M EDTA (Sigma Aldrich) and 10 μL of RNAseA (1.4 U/mL, Thermofisher scientific). Reagents were prepared according to manufacturer’s instructions. Standards were prepared using bacteriophage λ DNA (from the Cyquant kit) with a range from 10 ng/mL to 1.0 μg/mL of DNA. 50 μL of each standard was added in duplicate to a 96 well plate. Samples were assessed according to manufacturer’s instructions. Fluorescence was measured at excitation at 480 nm and emission 520 nm. DNA concentration was calculated by plotting DNA concentration versus the fluorescence of each sample, corrected for the background fluorescence.

#### Scanning electron microscopy

To visualise the ultrastructure of cell spheroids, cell-containing hydrogels were digested on day 10 using 0.5 mg/mL of collagenase (C2674-1G, Sigma-Aldrich, Wicklow, Ireland) for 3 h at 37°C, as previously described.^21^ All subsequent steps were performed in a fume hood. Samples were washed with PBS and fixed in 2% glutaraldehyde and 2% paraformaldehyde in 0.1 M sodium cacodylate buffer, pH 7.2, for 2 h at room temperature. Samples were dehydrated through a graded series of ethanol (30%, 50%, 70%, 90%, 100%) for 2 × 15 min. The GelMA spheroids were then placed in hexamethyldisilazane for 2 × 15 min. Following the final HMDS dehydration steps, the spheroids were gently centrifuged, excess HMDS was removed, and the spheroids were re-suspended in 200 μL of fresh HMDS. The spheroid suspension was then pipetted onto a glass slide and left to air-dry overnight. The glass slide was then mounted onto an aluminum SEM stub using a double-sided carbon tab and gold sputter-coated (Quorum Q150R ESplus). Samples were imaged on the Hitachi S-2600N scanning electron microscope (Hitachi, Krefeld, Germany).

#### Transmission electron microscopy

Day 10 gels were placed in 2% glutaraldehyde and 2% paraformaldehyde in 0.1 M sodium cacodylate buffer, pH 7.2, for 2 h at room temperature as previously described.^21^ Then, samples were placed in secondary fixative (1% osmium tetroxide) for 2 h prior to processing. Samples were dehydrated through a graded series of ethanol (30%, 50%, 70%, 90%, 100%) for 2 × 15 min followed by acetone for 2 × 20 min. The samples were then gradually infiltrated with resin (Agar Low-Viscosity Resin AGR1078) by placing the gels in a 50:50 resin and acetone mixture for 4 h, then a 75:25 resin and acetone mixture overnight and 100% resin for 6 h. Samples were transferred to an embedding mold filled with fresh 100% resin and then polymerised at 65°C for 48 h. Afterward, 500 nm semi-thin sections were cut using a glass knife, transferred onto a glass slide, stained with toluidine blue and viewed using a light microscope. From areas of interest, 70–90 nm ultrathin sections were cut using a diamond knife on a Lecia UC6 ultramicrotome, placed on 3 mm copper grids and allowed to air-dry. Sections were stained with UA-Zero EM stain (Agar) for 10 min and imaged on the Hitachi-7500 transmission electron microscope.

#### Immunohistochemical analysis of human colorectal cancer samples

Sections were taken from formalin-fixed paraffin-embedded (FFPE) tumor tissue blocks cut 3-5 μm thick with a rotary microtome. Sections were stained with haematoxylin and eosin (Diapath), CD68 (Clone PG-M1, Dako) and CD163 (Clone D6U1J, Cell Signaling) using benchtop staining or with a clinically validated automated Ventana benchmark ultra platform (Roche Diagnostics). Positive CD68 and CD163 cell quantification in stroma and tumor cells was subsequently carried out using open source QuPath software in a 10× field with standard settings for stains and with a threshold sensitivity of 0.3. The identification of tumor/stroma was done manually by a consultant histopathologist prior to quantification. Four or five separate representative fields per section were assessed for both tumor and stroma.

#### Transwell migration assay

Migration of THP-1 monocytes was analyzed using a Transwell migration assay. THP-1 cells were incubated in serum free media for 16 h prior to use. Conditioned medium from TCS or iTCS-treated hMSCs was placed at the bottom of a 24-well plate. THP-1 cell suspensions (1x10^5^ cells) were added into Transwell inserts (8 μm pore size) (Greneir Bio-one) and placed into the 24-well plate. The plate was returned to the incubator at 37°C for 4 h. Cells recovered from the lower compartment containing migrated THP-1 cells were collected and counted using the Cytek Northern Lights flow cytometer. Cell number was reported as absolute counts.

#### Subcutaneous tumor model

Animals were subcutaneously inoculated with 5 × 10^5^ CT26 cells ±1.5 × 10^5^ TCS or iTCS-treated MSCs in a total volume of 100 μL of PBS in the right flank. Animal welfare and tumour growth were monitored daily until experimental endpoint. Tumours were excised and harvested on day 13 post-injection.

#### Flow cytometry analysis of tumors

For TME studies, tumors were isolated from right flanks and processed by physical digestion followed by chemical digestion using a mouse Tumor Dissociation Kit (Miltenyi Biotech, Surrey, UK) according to the manufacturer’s protocol with some modifications. Incubation time was reduced to 2 h and dissociation was achieved by inverting the suspensions 10 times every 30 min. Tumors were processed further into single-cell suspensions using 40 μm cell strainers (ThermoFisher Scientific) and resuspended in dPBS. The single cell suspensions were then centrifuged at 400 × g for 5 min, resuspended and counted. 1 × 10^5^ cells per sample (and appropriate fluorescence minus one (FMO) controls) were stained with anti-mouse antibodies in fluorescence-activated cell sorting (FACS) buffer (dPBS supplemented with 1% FBS and 0.05% sodium azide): CD45, CD11b, MHC-II and CD206 (all Biolegend, CA, USA) (see key resources table for antibody catalog numbers). Viability was assessed with the cell viability dyes SYTOX Blue or SYTOX AADvanced (ThermoFisher Scientific). Surface staining was performed in FACS Buffer incubated at 4°C for 20 min, washed and resuspended in FACS buffer. Samples were acquired on the Cytek Northern Lights Flow Cytometer (Cytek Biosciences, CA, USA). Data was analyzed using FlowJo analysis software version 10 (Tree Star Inc., OR, USA) for intratumoral immune cells.

#### Flow cytometry analysis of stromal - immune cell co-cultures

Cells were collected at the endpoint of each experiment. 1 × 10^5^ cells/sample were stained with CD47, CD274, PD-L1, PD-L2 antibodies as detailed in the key resources table. Cell viability was assessed using SYTOX Blue (Thermo Fisher Scientific) or Zombie Violet for fixed panels. To assess cell viability in GelMA model, GelMA gels were dissociated by incubating gels with collagenase solution for 1.5 h at 37°C, followed by 0.25% trypsin-EDTA (Thermo Fisher Scientific) for 10 min. Viability was assessed using Sytox blue. Following co-culture, immune cells were incubated with TruStain FcX PLUS Fc block (Biolegend) prior to staining. For CAF co-cultures, 1 × 10^5^ cells were stained with CD11b, CD14, HLA-DR, PD-L1 and Sytox Blue to assess monocyte/macrophage phenotype after co-culture with CAFs and HCT116 cells. Cells were acquired on a BD FACSCanto II flow cytometer (BD Biosciences, San Jose, CA, USA or on the Cytek Northern Lights Flow Cytometer (Cytek Biosciences, CA, USA). Flow cytometry data were analyzed using FlowJo analysis software version 10 (Tree Star, Ashland, OR, USA).

#### Macrophage and MSC co-cultures, phagocytosis and antigen uptake assays

Murine BMDMs were plated at 5 × 10^5^ cells/well in 96-well flat bottom plates and activated by the addition of 100 ng/mL IFN-γ (Peprotech) and 10 ng/mL LPS (Sigma) as described above. The cells were washed with dPBS and 1 × 10^4^ mMSCs or conditioned mMSCs were added and cultured for a further 48 h. BMDMs were analyzed directly by flow cytometry or used in phagocytosis assays with cancer cells. For phagocytosis assays, 24 h after the addition of the mMSCs to the BMDM co-culture, CT26 cells were labeled with CellTrace CSFE (Thermo Fisher Scientific) and heated at 65°C for 10 min and 2.5 × 10^4^ CT26 cancer cells were added to each well. Phagocytosis was assessed after 24 h by flow cytometry. For the antigen uptake assay, 0.2 μg/mL DQ-Ovalbumin (Thermo Fisher Scientific) was added to wells 48 h after addition of mMSCs. Cells were incubated for 1 h at either 37°C or 4°C before analysis by flow cytometry. For antibody-blocking experiments, 10 μg/mL of each antibody or isotype control was added to the macrophages in 96-well plates for 30 min at room temperature in the dark, prior to the addition of the mMSCs. Fresh or conditioned mMSCs in 100 μL of MSC media was added to relevant wells and the experiment was conducted as described above.

#### PBMC:CAF co-culture

Tumor conditioned and control CAFs were plated at a density of 7.5 × 10^4^ CAFs per well of a 6-well plate in 2 mL of complete culture medium. 24 h later, medium was removed and replaced with 60% TCS or iTCS in fresh media and cultured for another 24 h. Conditioned CAFs were then co-cultured with 2.5 × 10^5^ PBMCs isolated from healthy patients for 24 h. Following this, 2.5 × 10^4^ heat-damaged HCT116 cells were added to the culture for a further 24 h. Primary monocytes/macrophages were assessed by flow cytometry by gating on CD14 and CD11b positive cells.

### Quantification and statistical analysis

Statistical analysis was performed using GraphPad Version 9 (La Jolla, CA, USA). Data were assessed for normal distribution using the D’Agostino–Pearson omnibus normality test where appropriate. Datasets with two groups were analyzed using paired and unpaired T-tests, where appropriate and as indicated in the figure legend. Datasets with more than two groups were analyzed by ordinary one-way ANOVA followed by the Tukey, Šídák’s or Dunnett’s multiple comparison tests. Data for individual animals and independent experiments are presented as individual symbols, unless otherwise stated. Tests for statistical differences between survival curves were performed using the default log rank test. Unless otherwise indicated in the figure legends, the data was plotted as mean ± SD to determine statistically significant differences. All statistical methods can also be found in the figure legends. Results were considered statistically significant at *p* < 0.05, indicated on graphs as ∗*p* < 0.05, ∗∗*p* < 0.01 and ∗∗∗*p* < 0.001. All statistically significant groups are indicated on graphs.
