# Supplementary material for: Tumor-associated mesenchymal stromal cells modulate macrophage phagocytosis in stromal-rich colorectal cancer via PD-1 signaling
Source: iScience. 2024 Aug 22;27(9):110701. doi: 10.1016/j.isci.2024.110701 (PMC11416555; doi:10.1016/j.isci.2024.110701)
Supplement: Document S1. Figures S1–S8 [file mmc1.pdf]

iScience, Volume *27*

Supplemental information

Tumor-associated mesenchymal stromal cells modulate macrophage phagocytosis in stromal-rich colorectal cancer via PD-1 signaling

Niamh A. Leonard, Shania M. Corry, Eileen Reidy, Hannah Egan, Grace O'Malley, Kerry Thompson, Emma McDermott, Aoise O'Neill, Norashikin Zakaria, Laurence J. Egan, Thomas Ritter, Daniela Loessner, Keara Redmond, Margaret Sheehan, Aoife Canney, Aisling M. Hogan, Sean O. Hynes, Oliver Treacy, Philip D. Dunne, and Aideen E. Ryan

Supplementary Figure S1. Chemokine expression differs between the stromal and epithelial compartments of CRC (related to Figure 1). ***a*** Expression boxplots of chemoattractant gene transcripts in the stromal compartment and epithelial compartments of CRC tumours (n=13) generated by ConfoundR (https://confoundr.qub.ac.uk/) using dataset GSE35602. Wilcoxon rank sum test. ***b*** Gene set enrichment analysis (GSEA) of KEGG and Hallmark gene sets between the stromal and epithelial compartments using ConfoundR (GSE35602).

Supplementary Figure S2. Quantification of CD68+ and CD163+ macrophages in the stromal and epithelial compartments of CRC (related to Figure 4). ***a*** CD68 and ***b*** CD163 staining of primary colorectal tumour sections was conducted using QuPath. 4-5 fields of view were analysed per section. Areas outlined in red denotes the epithelium, while areas outlined in blue/yellow denotes the stroma. ***c*** Secondary only control staining of colorectal tumour sections. Representative images displayed, scale bar =100μm or 50μm.

Supplementary Figure S3. Enrichment of macrophage-related pathways across consensus molecular subtypes (related to Figure 4). Heatmap of single sample gene set enrichment analysis (ssGSEA) scores of gene signatures related to macrophage-related pathways (GSE39582), separated by CMS classification (n=258; CMS1 = 49, CMS2 = 75, CMS3 = 35, CMS4 = 58, unknown = 41).

Supplementary Figure S4. PD-L1/CD274 transcript expression is higher in the stromal compartment of CRC (related to Figure 5). Expression boxplots of *CD47* and *CD274* gene transcripts in the stromal and epithelial compartments of CRC tumours (n=13) generated by ConfoundR (GSE35602). Wilcoxon rank sum test.

Supplementary Figure S5. PD-L1 and CD47 expression on MSCs is actively induced by iTCS (related to Figure 5). Expression of (***a***) CD47 and (***b***) PD-L1 on Balb/c MSCs treated with CT26 cancer cell media, TCS, iTCS, recombinant TNFα (100ng/ml) or heat inactivated (HI) TCS and iTCS (n=3). Data displayed as MFI. Error bars, mean ± SD; *, P < 0.05, **** P < 0.0001 by one-way ANOVA and Tukey post hoc test.

Supplementary Figure S6. Characterisation of the human MSC response to TCS or iTCS and characterisation of *ex vivo* CAF phenotypes (Related to Figure 5). ***a*** CD47 and PD-L1 expression on human bone marrow-derived MSCs following treatment with TCS or iTCS generated from HT29 or HCT116 human CRC cell lines. Data analysed by flow cytometry and displayed as MFI. ***b*** Histograms showing positive expression of the CAF markers CD90, FAP, Podoplanin and PDGFR-α and lack of expression of leukocyte (CD45) and epithelial (EpCAM) markers. CAFs were isolated from primary human CRC tumours. Error bars, mean ± SD; *, P < 0.05, **, P < 0.01, ***, P < 0.001 by one-way ANOVA and Tukey post hoc test.

Supplementary Figure S7. MSCs reduce macrophage OVA protein uptake and processing (related to Figure 7). ***a*** Experimental outline. Balb/c-derived BMDMs and MSC co-cultures were treated with DQ-Ovalbumin to assess antigen uptake and processing. Flow cytometry analysis of processed DQ-Ovalbumin measured as MFI in (***b***) naïve and (***c***) IFN-γ+LPS activated macrophages following co-culture with control or tumour-conditioned MSCs. ***d*** Representative histograms of CD11b+ macrophages after co-culture with MSCs and addition of DQ-Ova. (n=3). Error bars, mean ± SD; **, P < 0.01; ***, P < 0.001; ****, P < 0.0001 by one-way ANOVA and Tukey post hoc test.

Supplementary Figure S8. SIRPα and PD-1 blocking antibodies do not alter macrophage phagocytosis when added to macrophages cultured alone or with fresh or TCS MSCs (Related to Figure 8). ***a*** Experimental outline depicting naïve or IFN-γ+LPS activated macrophage phagocytosis of fluorescently labelled (CFSE) cancer cells in the presence of SIRPα or PD-1 blocking antibodies. ***b*** Naïve or IFN-γ+LPS activated macrophages were cultured alone or in the presence of SIRPα (left) or (right) PD-1 blocking monoclonal antibodies or relevant isotype controls. Data shows relative frequency (relative to macrophages alone) of CFSE-expressing CD11b+ macrophages following internalisation of CFSE-labelled cancer cells. ***c*** Same experimental setup as shown in ***a*** with the addition of co-culture of macrophages with TCS-conditioned MSCs. ***d*** Naïve or IFN-γ+LPS activated macrophages were cultured alone or co-cultured with TCS-conditioned MSCs in the presence of SIRPα (left) or (right) PD-1 blocking monoclonal antibodies or relevant isotype controls. Data shows relative frequency (relative

to macrophages alone) of CFSE-expressing CD11b+ macrophages following internalisation of CFSE-labelled cancer cells. (n=4-8). Error bars, mean ± SD; *, $P < 0.05$, ***, $P < 0.001$ by one-way ANOVA and Tukey post hoc test.

Figure S1

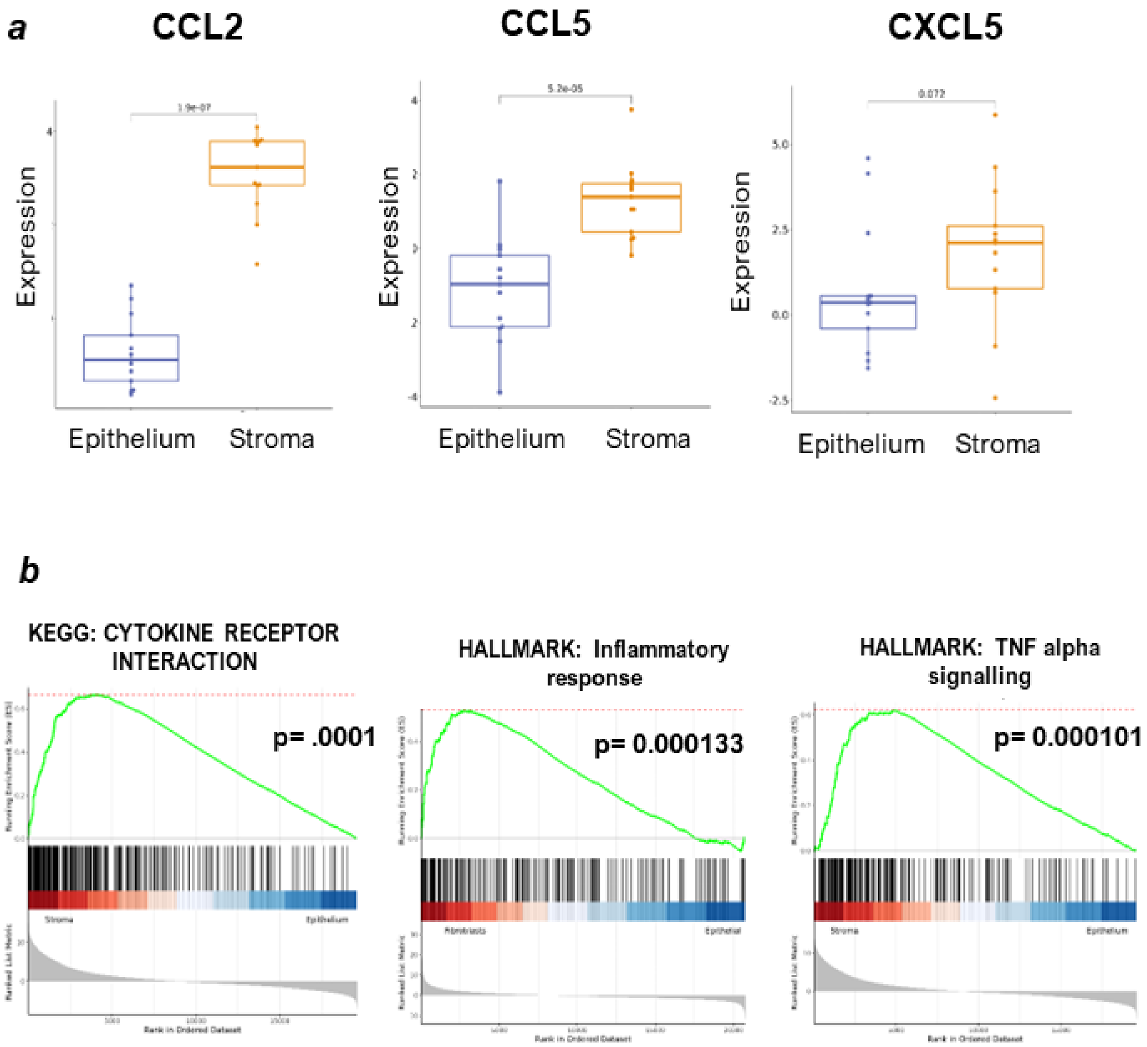

Figure S2

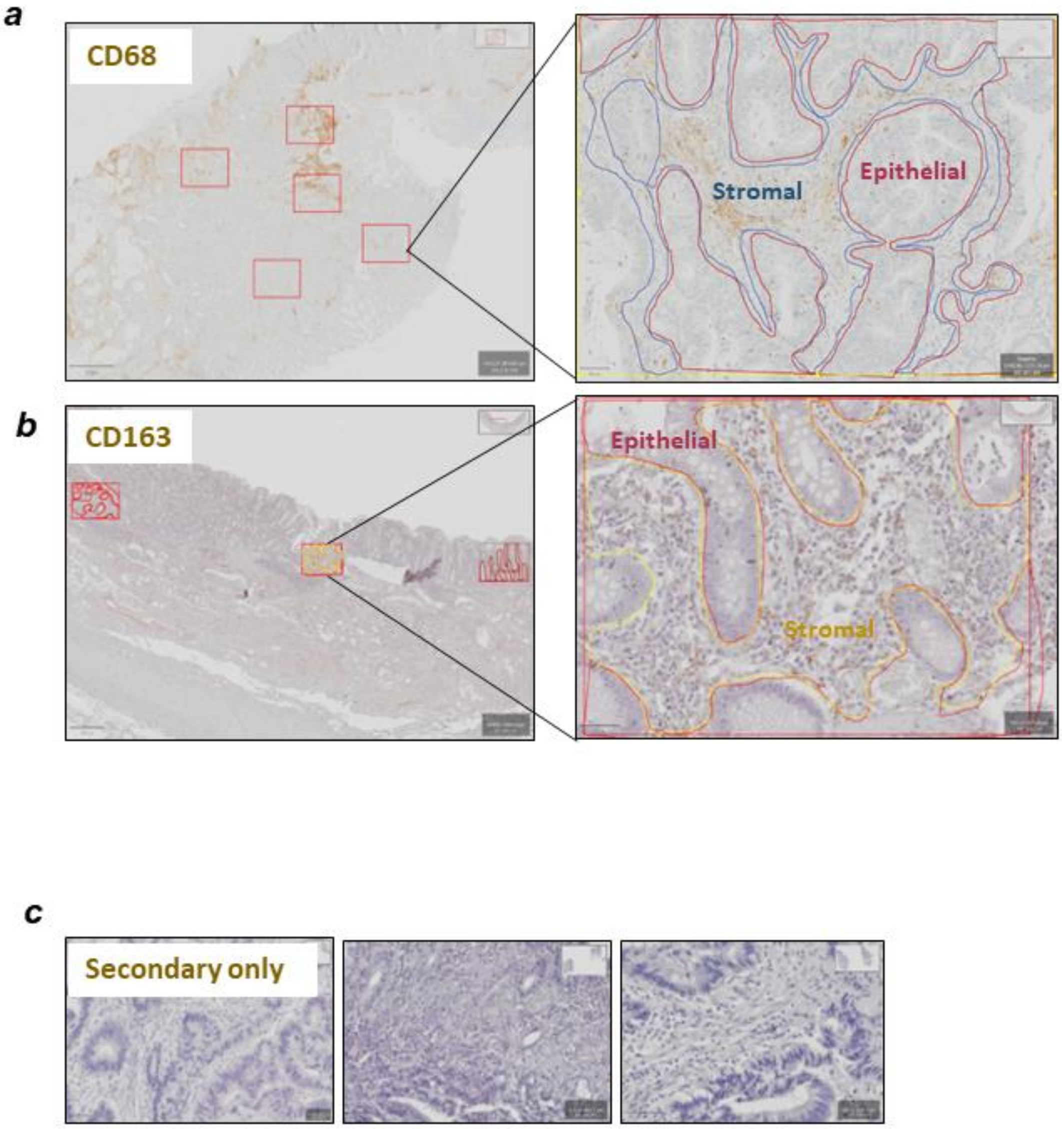
a
CD68
Stromal
Epithelial
b
CD163
Epithelial
Stromal
c
Secondary only

Figure S3

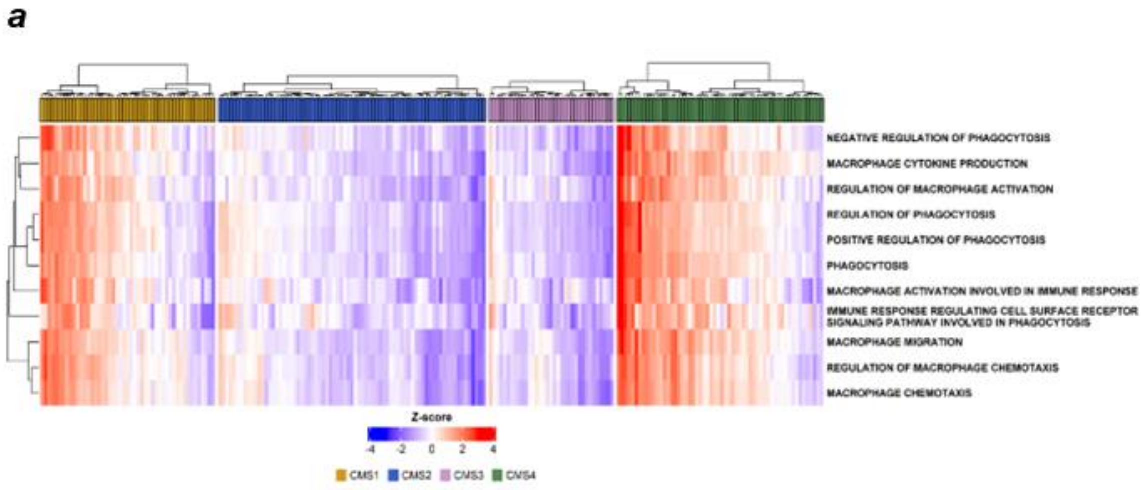

Figure S4

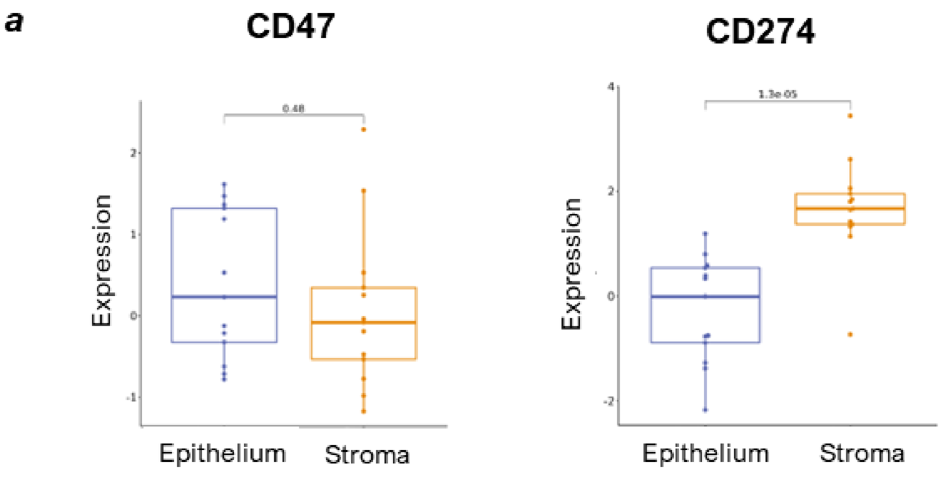

Figure S5

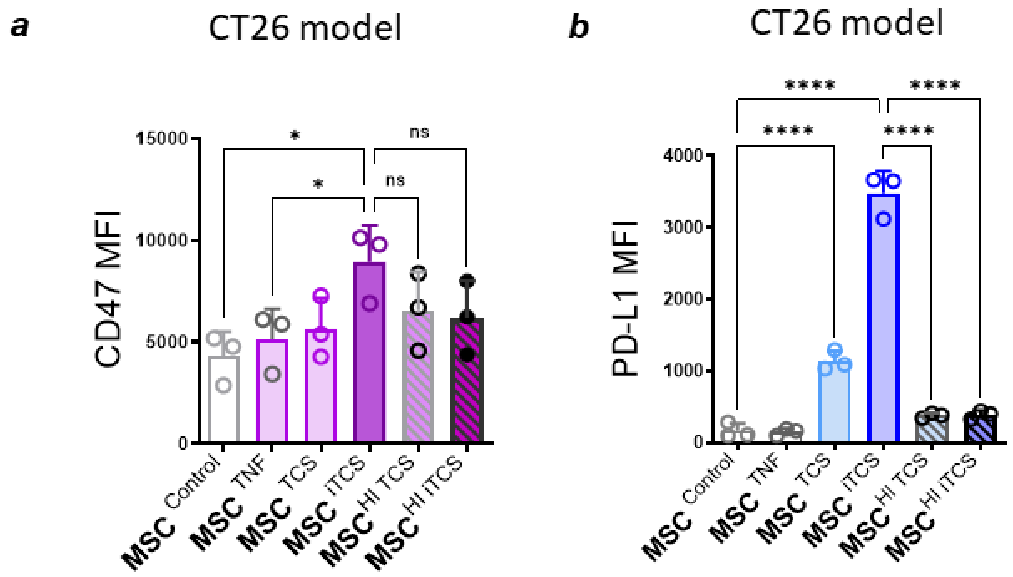
a
CT26 model
CD47 MFI
15000
10000
5000
0
*
*
ns
ns
MSC Control
MSC TNF
MSC TCS
MSC iTCS
MSC HI TCS
MSC HI iTCS
b
CT26 model
PD-L1 MFI
4000
3000
2000
1000
0
****
****
****
****
MSC Control
MSC TNF
MSC TCS
MSC iTCS
MSC HI TCS
MSC HI iTCS

Figure S6

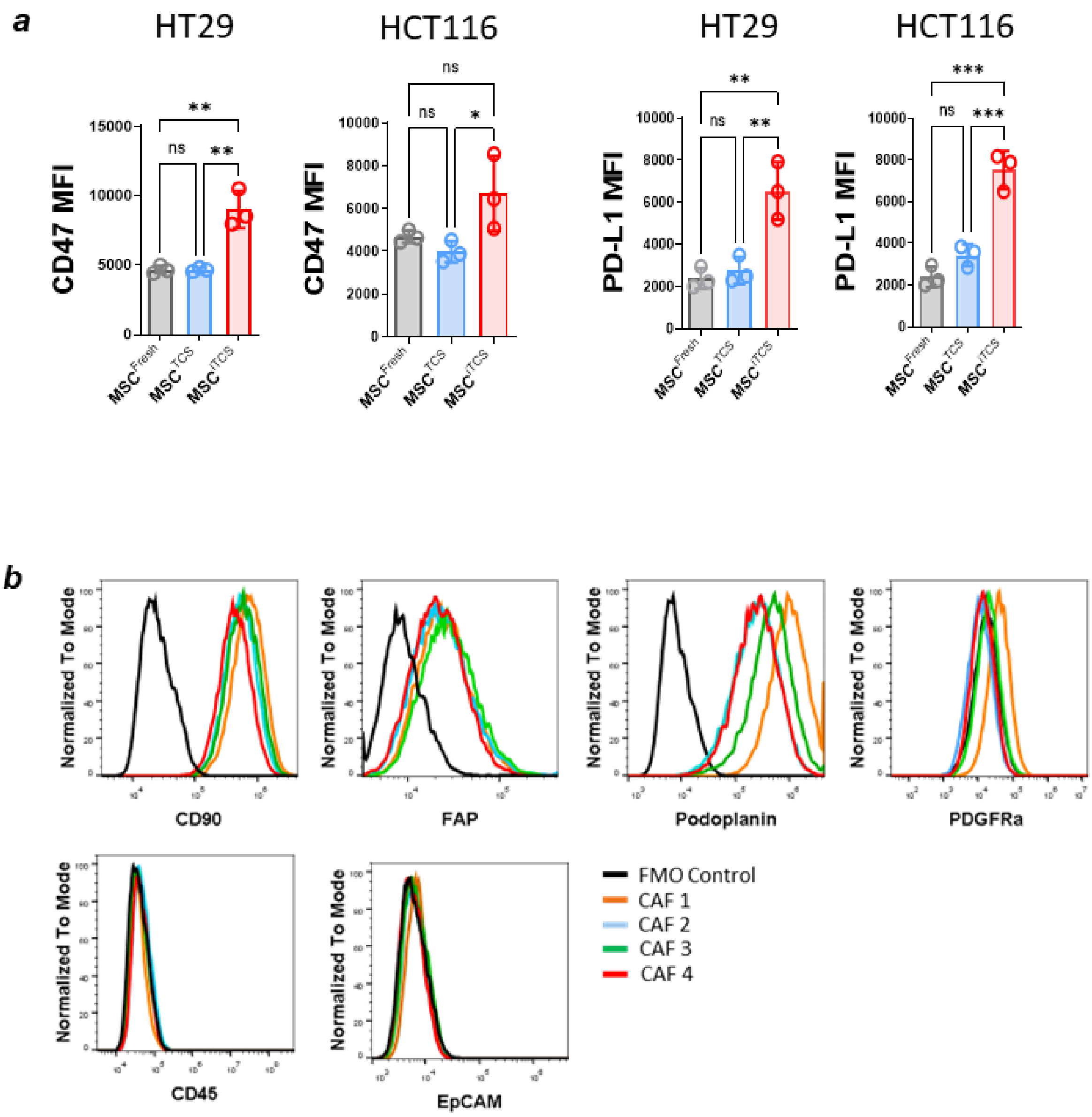

a
HT29
HCT116
HT29
HCT116
CD47 MFI
PD-L1 MFI
ns
*
**
***
MSC Fresh
MSC TCS
MSC iTCS
b
Normalized To Mode
CD90
FAP
Podoplanin
PDGFRa
CD45
EpCAM
FMO Control
CAF 1
CAF 2
CAF 3
CAF 4

Figure S7

a

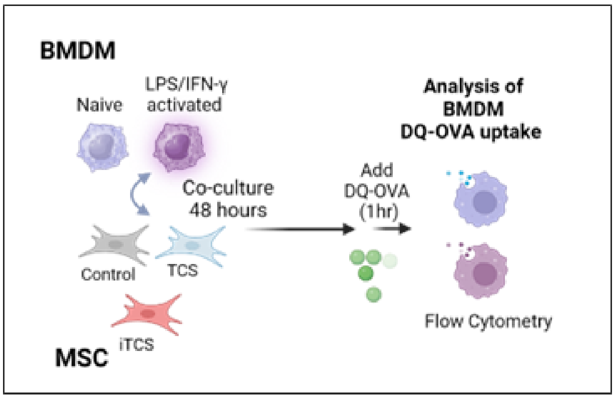

b

c

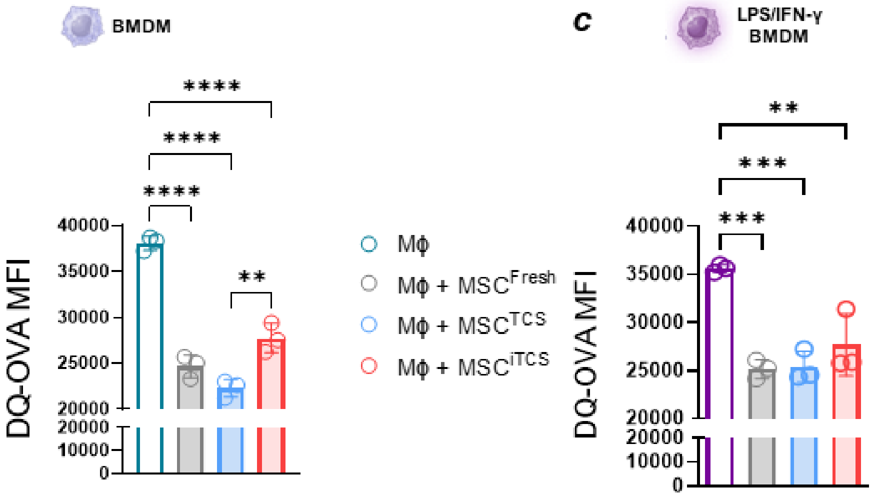

d

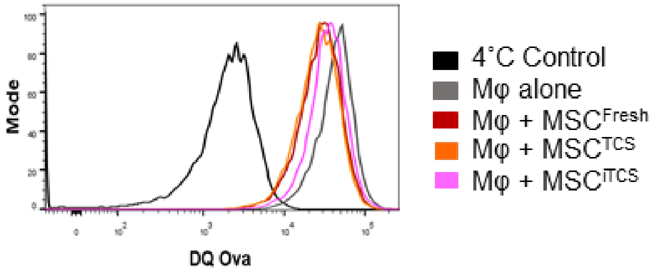

Figure S8

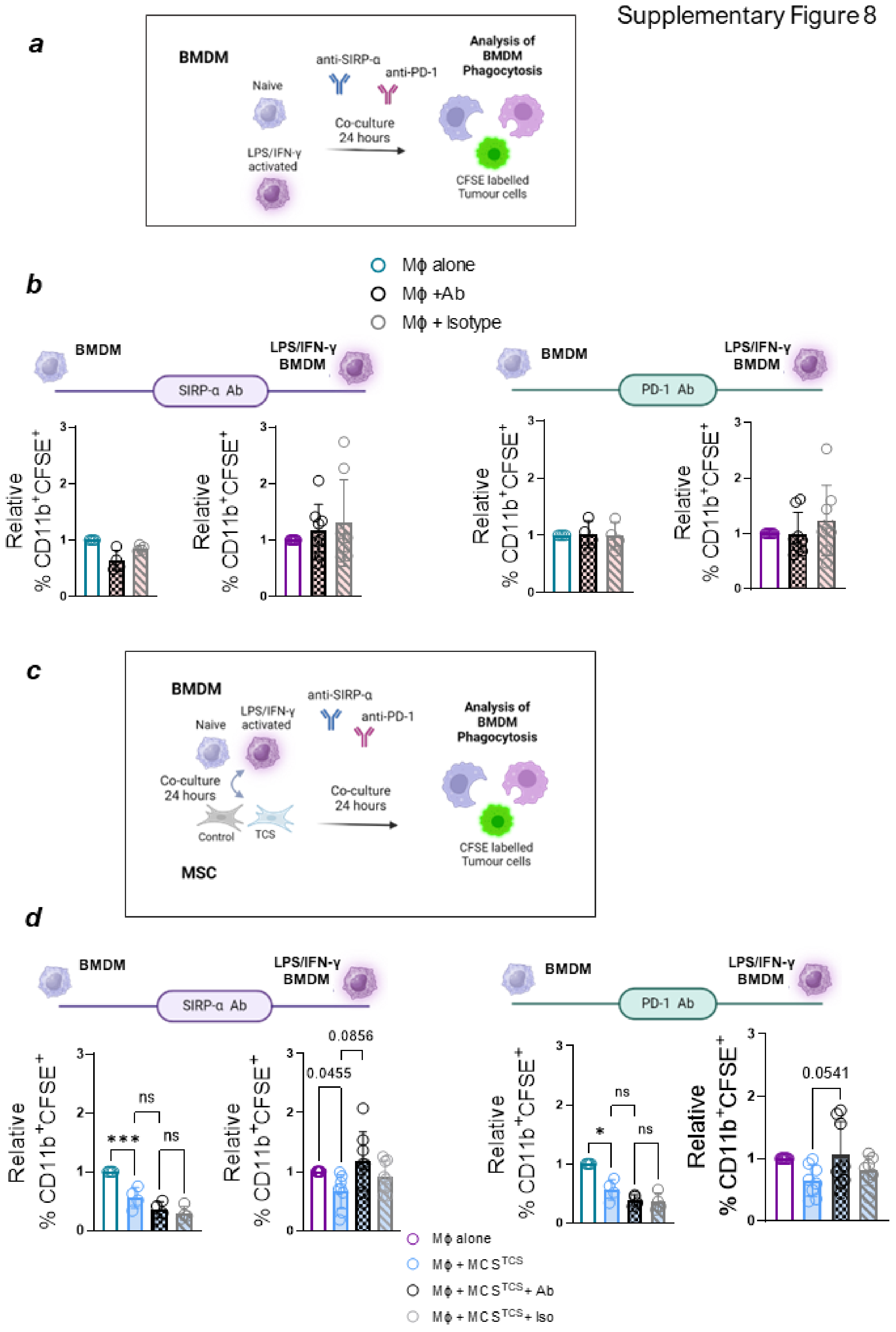

Supplementary Figure 8
a
BMDM
Naive
LPS/IFN-γ activated
anti-SIRP-α
anti-PD-1
Co-culture 24 hours
Analysis of BMDM Phagocytosis
CFSE labelled Tumour cells
b
Mϕ alone
Mϕ +Ab
Mϕ + Isotype
BMDM
LPS/IFN-γ BMDM
SIRP-α Ab
PD-1 Ab
Relative % CD11b+CFSE+
c
BMDM
Naive
LPS/IFN-γ activated
Co-culture 24 hours
Control
TCS
MSC
anti-SIRP-α
anti-PD-1
Co-culture 24 hours
Analysis of BMDM Phagocytosis
CFSE labelled Tumour cells
d
BMDM
LPS/IFN-γ BMDM
SIRP-α Ab
PD-1 Ab
Relative % CD11b+CFSE+
***
ns
0.0455
0.0856
*
0.0541
Mϕ alone
Mϕ + MCS TCS
Mϕ + MCS TCS + Ab
Mϕ + MCS TCS + Iso
